# Neoantigen quality predicts immunoediting in survivors of pancreatic cancer

**DOI:** 10.1038/s41586-022-04735-9

**Published:** 2022-05-19

**Authors:** Marta Łuksza, Zachary M. Sethna, Luis A. Rojas, Jayon Lihm, Barbara Bravi, Yuval Elhanati, Kevin Soares, Masataka Amisaki, Anton Dobrin, David Hoyos, Pablo Guasp, Abderezak Zebboudj, Rebecca Yu, Adrienne Kaya Chandra, Theresa Waters, Zagaa Odgerel, Joanne Leung, Rajya Kappagantula, Alvin Makohon-Moore, Amber Johns, Anthony Gill, Mathieu Gigoux, Jedd Wolchok, Taha Merghoub, Michel Sadelain, Erin Patterson, Remi Monasson, Thierry Mora, Aleksandra M. Walczak, Simona Cocco, Christine Iacobuzio-Donahue, Benjamin D. Greenbaum, Vinod P. Balachandran

**Affiliations:** 1 Tisch Cancer Institute, Departments of Oncological Sciences and Genetics and Genomic Sciences, Icahn School of Medicine at Mount Sinai, New York, NY USA; 2 Computational Oncology Service, Department of Biostatistics, Memorial Sloan Kettering Cancer Center, New York, NY USA; 3 Immuno-Oncology Service, Human Oncology and Pathogenesis Program, Memorial Sloan Kettering Cancer Center, New York, NY USA; 4 Hepatopancreatobiliary Service, Department of Surgery, Memorial Sloan Kettering Cancer Center, New York, NY USA; 5 Laboratoire de Physique de l’Ecole Normale Supérieure, ENS, Université PSL, CNRS, Sorbonne Université, Université de Paris, Paris, France; 6 Department of Mathematics, Imperial College London, London, UK; 7 David M. Rubenstein Center for Pancreatic Cancer Research, Memorial Sloan Kettering Cancer Center, New York, NY USA; 8 Center for Cell Engineering, Memorial Sloan Kettering Cancer Center, New York, NY USA; 9 Immunology Program, Sloan Kettering Institute, Memorial Sloan Kettering Cancer Center, New York, NY USA; 10 Human Oncology and Pathogenesis Program, Memorial Sloan Kettering Cancer Center, New York, NY USA; 11 The Kinghorn Cancer Centre, Garvan Institute of Medical Research, Darlinghurst, New South Wales Australia; 12 University of Sydney, Sydney, New South Wales Australia; 13 Swim Across America and Ludwig Collaborative Laboratory, Parker Institute for Cancer Immunotherapy, Memorial Sloan Kettering Cancer Center, New York, NY USA; 14 Physiology, Biophysics & Systems Biology, Weill Cornell Medicine, Weill Cornell Medical College, New York, NY USA; 15 Parker Institute for Cancer Immunotherapy, Memorial Sloan Kettering Cancer Center, New York, NY USA

**Keywords:** Immunosurveillance, Cancer genomics, Computational biophysics

## Abstract

Cancer immunoediting^1^ is a hallmark of cancer^2^ that predicts that lymphocytes kill more immunogenic cancer cells to cause less immunogenic clones to dominate a population. Although proven in mice^1,3^, whether immunoediting occurs naturally in human cancers remains unclear. Here, to address this, we investigate how 70 human pancreatic cancers evolved over 10 years. We find that, despite having more time to accumulate mutations, rare long-term survivors of pancreatic cancer who have stronger T cell activity in primary tumours develop genetically less heterogeneous recurrent tumours with fewer immunogenic mutations (neoantigens). To quantify whether immunoediting underlies these observations, we infer that a neoantigen is immunogenic (high-quality) by two features—‘non-selfness’  based on neoantigen similarity to known antigens^4,5^, and ‘selfness’  based on the antigenic distance required for a neoantigen to differentially bind to the MHC or activate a T cell compared with its wild-type peptide. Using these features, we estimate cancer clone fitness as the aggregate cost of T cells recognizing high-quality neoantigens offset by gains from oncogenic mutations. With this model, we predict the clonal evolution of tumours to reveal that long-term survivors of pancreatic cancer develop recurrent tumours with fewer high-quality neoantigens. Thus, we submit evidence that that the human immune system naturally edits neoantigens. Furthermore, we present a model to predict how immune pressure induces cancer cell populations to evolve over time. More broadly, our results argue that the immune system fundamentally surveils host genetic changes to suppress cancer.

## Abstract (web summary)

The human immune system naturally edits cancers of high-quality neoantigens.

## Main

In 1957, Burnet and Thomas proposed that the immune system in multicellular organisms must eliminate transformed cells as an evolutionary necessity to maintain tissue homeostasis. This theory of ‘cancer immunosurveillance’ was later redefined more broadly as ‘cancer immunoediting’^6^—as a consequence of the immune system protecting the host from cancer, the immune system must also sculpt developing cancers^1,7^. When cancers develop, they accumulate mutations, some of which generate new protein sequences (neoantigens)^8^. As neoantigens are mostly absent from the human proteome, they can escape T cell central tolerance in the thymus to become antigens in cancers^8^. However, neoantigens typically arise in passenger mutations, and therefore distribute heterogeneously in cancer cell clones with variable immunogenicity. Thus, T cells selectively ‘edit’ clones^1^ with more immunogenic neoantigens^3^, inducing less immunogenic clones to outgrow in cancers.

Although cancer immunoediting has been demonstrated through longitudinal studies in immune-proficient and immune-deficient mice^1,3,8^, whether it is a general principle of how human cancers evolve remains uncertain. Despite suggestive evidence^9–11^, definitive evidence requires longitudinal tracking of large numbers of patients and cancers over time. As this is logistically challenging, whether the human immune system naturally edits cancers and whether edited clones can be predicted a priori remain unclear.

## Quantifying selection pressures on neoantigens

**Fig. 1:**
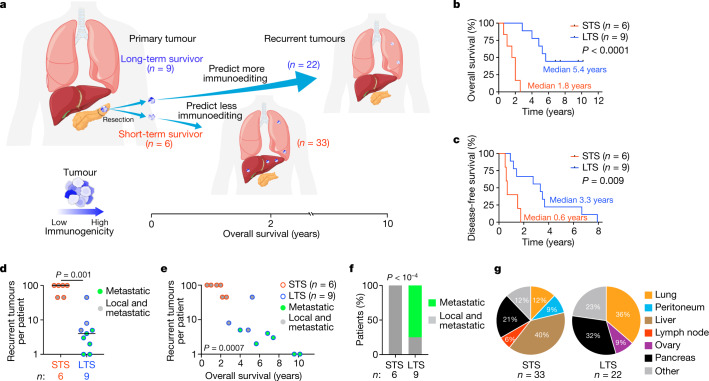
LTSs of PDAC develop tumours with distinct recurrence time, multiplicity and tissue tropism. **a**, The experimental design. **b**, **c**, Overall survival (**b**) and disease-free survival (**c**) of patients with PDAC. **d**–**g**, The number (**d**), correlation with overall survival (**e**), patterns (**f**) and sites (**g**) of recurrent PDACs. In **g**, other indicates omentum, aorta, diaphragm and perirectum (STS); and pericardium, inferior vena cava, adrenal, kidney and liver (LTS). *n* indicates the number of individual patients (**b**–**f**) or recurrent tumours (**g**). The horizontal bars show the median values. *P* values were determined using two-tailed log-rank tests (Mantel–Cox; **b** and **c**), two-tailed Mann–Whitney *U*-tests (**d**), two-tailed Pearson correlation (**e**) and two-tailed *χ*^2^ tests (**f**). Source data

To address this, we examined how 70 pancreatic ductal adenocarcinomas (PDACs) from 15 patients evolved longitudinally over 10 years (Fig. 1a). We reasoned that PDAC is an ideal cancer to test the immunoediting hypothesis. First, human PDACs have fewer neoantigens (35 on average)^5,12^ compared with more immunogenic cancers (112 in non-small-cell lung cancer^13^, 370 in melanoma^14^ on average). This theoretically maximizes our ability to both distinguish true neoantigen selection from neutral genomic changes over time and isolate effects of individual neoantigens on clonal selection. Second, T cell infiltrates in PDACs range from nearly zero to 1,000-fold higher^5^. Thus, PDACs have subsets that approximate immune-deficient and immune-proficient cancers, enabling us to theoretically observe how differential immune selection pressures modulate cancer cell clones. Finally, mutations in oncogenes occur early in PDAC carcinogenesis and are clonal^15^—this largely equalizes the cell-intrinsic oncogenic pressures among clones, maximizing our ability to detect how cell-extrinsic immune pressures affect clonal evolution.

**Extended Data Fig. 1:**
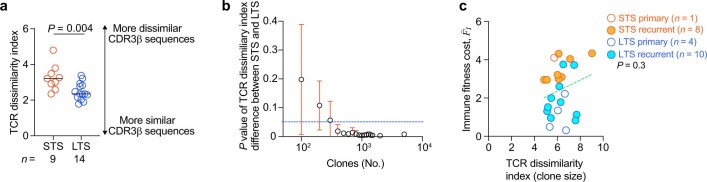
Top ranked T cells in LTS tumours have more similar CDR3β sequences. (**a**) T cell receptor (TCR) CDR3β sequence dissimilarity (TCR dissimilarity index) in STS and LTS primary and recurrent PDACs. TCR dissimilarity index calculated using the Restricted Boltzmann Machine model^18^. *n* = individual tumours. Horizontal bars = median. (**b**) Trend of *P* value of TCR dissimilarity index between STS and LTS PDACs (as in left panel) with number of clones in the sample. *n* = 17 tumours. Blue line indicates a *P* value of 0.05; circle = mean *P* value; error bars = standard error of the mean. (**c**) TCR dissimilarity index based on T cell clone size (Supplementary Methods) and immune fitness cost ${\bar{F}}_{I}$. Green line = linear regression fit. *P* value by two-tailed Mann-Whitney *U*-test (**a**) and two-tailed Pearson correlation (**c**). Source data

To model how immune-proficient and immune-deficient human cancers evolve, we compared how primary PDACs evolve to recurrence in a cohort of long-term survivors (LTSs) and short-term survivors (STSs) (Fig. 1a, b and Supplementary Table 1). We previously demonstrated that, compared with STSs, LTSs have primary tumours with around a 12-fold greater number of activated CD8^+^ T cells^5,16,17^ that are predicted to target immunogenic neoantigens^5^, therefore phenocopying relative greater immune pressure. Furthermore, in the current cohort we find that the largest T cell clones of LTS tumours have more similar CDR3β sequences^18^ compared with the largest T cell clones in STS tumours (Extended Data Fig. 1a, b), suggesting T cell clonal expansion and therefore greater immune activity in LTSs. We therefore hypothesized that this higher immune pressure in LTSs would induce tumours to preferentially lose tumour clones with immunogenic neoantigens over time (Fig. 1a). To test this hypothesis, we compared how tumours evolved from primary to recurrent tumours. We found that compared with STSs, LTSs had later (Fig. 1c) and fewer recurrent tumours (Fig. 1d) that inversely correlated with longer survival times (Fig. 1e). Moreover, 75% of LTSs versus 0% of STSs had recurrent tumours that were only metastatic (Fig. 1f), with distinct tissue-tropic recurrence patterns (Fig. 1g). Thus, LTS tumours recur with distinct latency, multiplicity and tissue-dependent evolutionary trajectories.

**Fig. 2:**
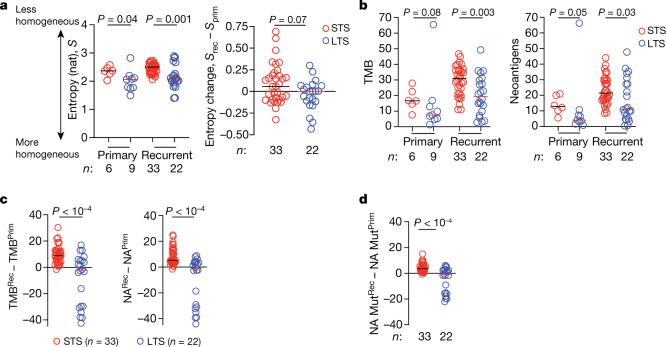
LTSs of PDAC develop tumours with fewer neoantigens. **a**, Shannon entropy (*S*, left), and the difference in Shannon entropy between recurrent (*S*_rec_) and primary (*S*_prim_) PDACs (right). **b**, TMB and neoantigen number (NA) in primary and recurrent PDACs. **c**, **d**, The difference in TMB and NA (**c**), and the number of mutations that generate neoantigens (NA Mut) (**d**) between recurrent and primary PDACs. *n* indicates the number of individual tumours. The horizontal bars show the median values. For **a**–**d**, *P* values were determined using two-tailed Mann–Whitney *U*-tests. Source data

**Extended Data Fig. 2:**
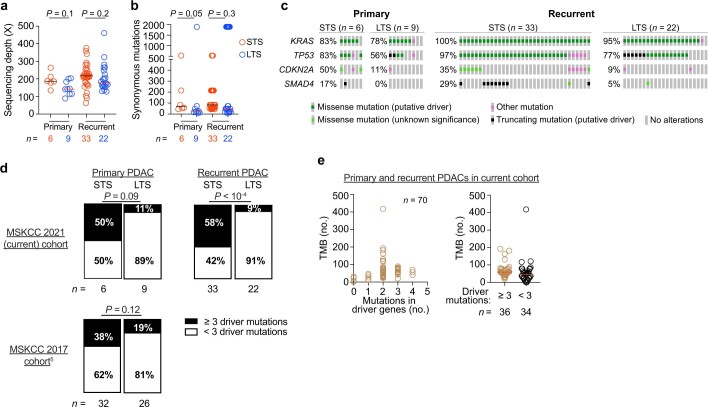
Tumour mutational features in STSs and LTSs of PDAC . (**a**) Whole-exome sequencing depth and (**b**) number of synonymous mutations in primary and recurrent PDACs from STSs and LTSs. (**c**) Oncoprints of driver mutation frequencies in primary and recurrent PDACs. Frequencies = percentage of patients in each cohort that harbor corresponding driver gene mutations. (**d**) Frequency of primary (left) and recurrent (right) PDACs with mutations in ≥ 3 oncogenes. (**e**) Number of nonsynonymous mutations (TMB) versus number of mutations in oncogenes in primary and recurrent PDACs. *n* = individual tumours. Horizontal bars = median. *P* value by two-tailed Mann-Whitney *U*-test. Source data

**Extended Data Fig. 3:**
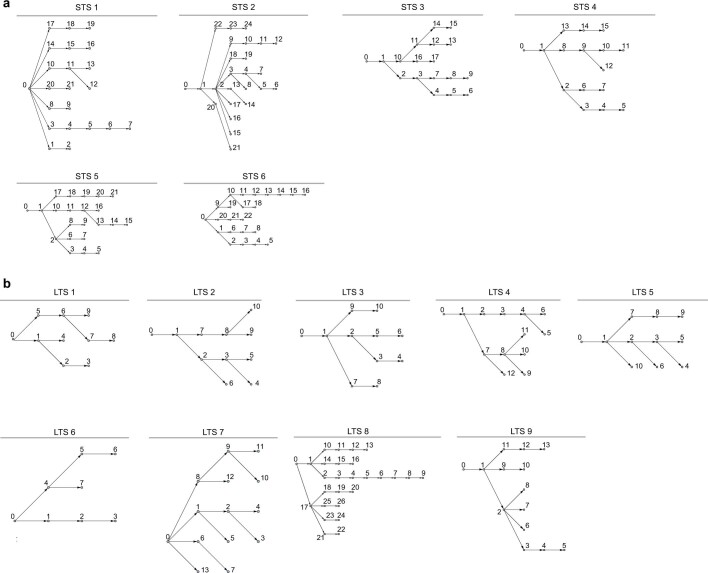
Tumour evolutionary trees in STSs and LTSs of PDAC. (**a**, **b**) Tumour clone phylogenies in primary and recurrent PDACs from STSs (**a**, *n* = 6) and LTSs (**b**, *n* = 9).

To examine whether differential selection pressure could explain these unique recurrence patterns, we performed whole-exome sequencing (Extended Data Fig. 2a) and inferred the clonal structures of matched primary and recurrent tumours. We reasoned that greater immune selection pressure in LTS tumours should limit the diversity of tumour clones over time, due to immunoediting of neoantigens. Consistently, we found that, although primary tumours in LTSs were only slightly more homogeneous than in STSs, recurrent tumours in LTSs were much more homogeneous (Fig. 2a (left)), indicating that LTSs probably evolved fewer clones (Fig. 2a (right) and Extended Data Fig. 3a, b). To examine whether this could be explained by greater selection pressure on neoantigens, we compared the total number of non-synonymous mutations (tumour mutational burden (TMB)) and computationally predicted MHC-I restricted neoantigens^4,5^. Consistently, although primary LTS tumours had a similar TMB with a comparable number of neoantigens as STS tumours (Fig. 2b), recurrent LTS tumours had a lower TMB with fewer neoantigens (Fig. 2b). Despite these differences, LTS and STS tumours had comparable numbers of synonymous mutations and mutations in driver oncogenes (Extended Data Fig. 2b, c). Although recurrent tumours of LTSs had fewer co-occurring mutations in oncogenes compared with recurrent tumours of STSs (Extended Data Fig. 2d), the number of mutations in oncogenes did not correlate with TMB (Extended Data Fig. 2e). Furthermore, LTS recurrent tumours gained significantly fewer mutations and neoantigens compared with STS recurrent tumours (Fig. 2c), remaining largely neutral over time^19^. LTS tumours also gained fewer mutations that generate neoantigens than STS tumours (Fig. 2d), indicating that LTS tumours preferentially depleted neoantigenic mutations. These data support the hypothesis that greater immune selection in LTS tumours edited tumour clones and neoantigens.

## The neoantigen quality model

**Fig. 3:**
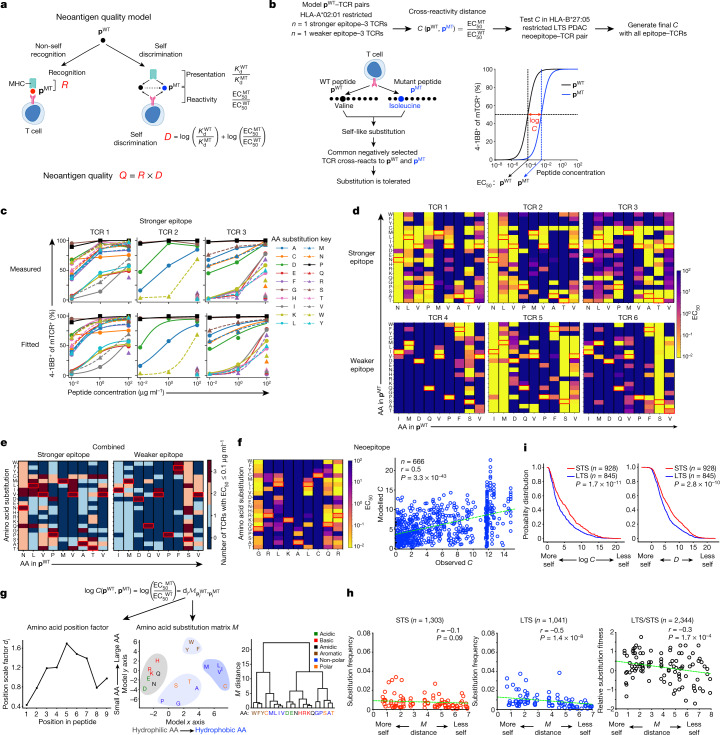
High-quality neoantigens are immunoedited in LTS  PDACs. **a**, Neoantigen quality model. **b**, The model and experimental approach to estimate cross-reactivity distance *C*. **c**, **d**, Measured (top) and fitted (bottom) **p**^MT^–TCR activation curves (**c**, amino acid (AA) position 4), and activation heat maps (**d**, all amino acid positions) for stronger and weaker **p**^WT^–TCR pairs. **e**, Composite **p**^MT^–TCR EC_50_ values of all stronger and weaker **p**^WT^–TCR pairs. **f**, **p**^MT^–TCR activation heat map and observed versus modelled *C*(**p**^WT^, **p**^MT^) for the HLA-B*27:05-restricted **p**^WT^–TCR pair. *n* indicates the number of single-amino-acid-substituted **p**^WT^, **p**^MT^ and **p**^MT^, **p**^MT^ pairs. **g**, Cross-reactivity distance model *C* and dendrogram of agglomerative clustering of substitution matrix *M*. **h**, Observed amino acid substitution frequency versus matrix *M*-defined substitution distance in primary and recurrent STS and LTS PDACs. *M* distance is the matrix *M*-defined amino acid distance from **g**. Circles indicate substituted residues. *n* indicates the number of substitutions**. i**, Cumulative probability distributions of log(*C*) and *D*. *n* indicates the number of neoantigens. The red rectangles in the heat maps indicate amino acids in **p**^WT^. The green line is a linear regression fit. Heat maps are ordered according to the amino acid order in the dendogram in **g**. *P* values were determined using two-tailed Pearson correlation (**f** and **h**) and two-sided Kolmogorov–Smirnov tests (**i**). Source data

To identify the edited neoantigens, we extended our previous neoantigen quality model^4,5^ that quantifies the immunogenic features of a neoantigen to propose that two competing outcomes determine whether a neoantigen is high-quality—whether the immune system recognizes or tolerates a neoantigenic mutation (Fig. 3a). To estimate the likelihood the immune system recognizes a neoantigen, we measure the sequence similarity of the mutant neopeptide (**p**^MT^) to known immunogenic antigens. This infers the ‘non-self’ recognition potential *R* of **p**^MT^, a proxy for peptides within the recognition space of the T cell receptor (TCR) repertoire.

By contrast, we posit that the immune system can also fail to discriminate **p**^MT^ from its wild-type (WT) peptide (**p**^WT^), and therefore tolerate it as ‘self’. The immune system must therefore exert greater self discrimination *D* (Fig. 3a) in tumours to overcome the principles of negative T cell selection, the adaptation that limits autoreactivity to host tissues. We approximate the *D* between **p**^WT^ and **p**^MT^ by two features—differential MHC presentation and differential T cell reactivity. Differential MHC presentation of **p**^WT^ and **p**^MT^ (${K}_{{\rm{d}}}^{\text{WT}}/{K}_{{\rm{d}}}^{\text{MT}}$), previously introduced as the MHC amplitude *A* (refs. ^4,5^), estimates the availability of T cells to recognize **p**^MT^. If **p**^WT^ is not presented to T cells in the thymus or the periphery (as with a high ${K}_{{\rm{d}}}^{\text{WT}}$, which implies poor **p**^WT^–MHC binding), **p**^WT^-specific T cells escape negative selection to expand the peripheral T cell precursor pool available to recognize a **p**^MT^ presented on MHC (low ${K}_{{\rm{d}}}^{\text{MT}}$)^20^. Here we extend this concept and introduce cross-reactivity distance *C*, a new model term that estimates the antigenic distance required for T cells to discriminate between **p**^MT^ and **p**^WT^. Thus, self discrimination *D* = log(*A*) + log(*C*) is a proxy for peptides outside the toleration space of the TCR repertoire. In summary, we define neoantigen quality as *Q* = *R *× *D* (Fig. 3a), now with components that estimate whether a neoantigen can be recognized as non-self and discriminated from self.

**Extended Data Fig. 4:**
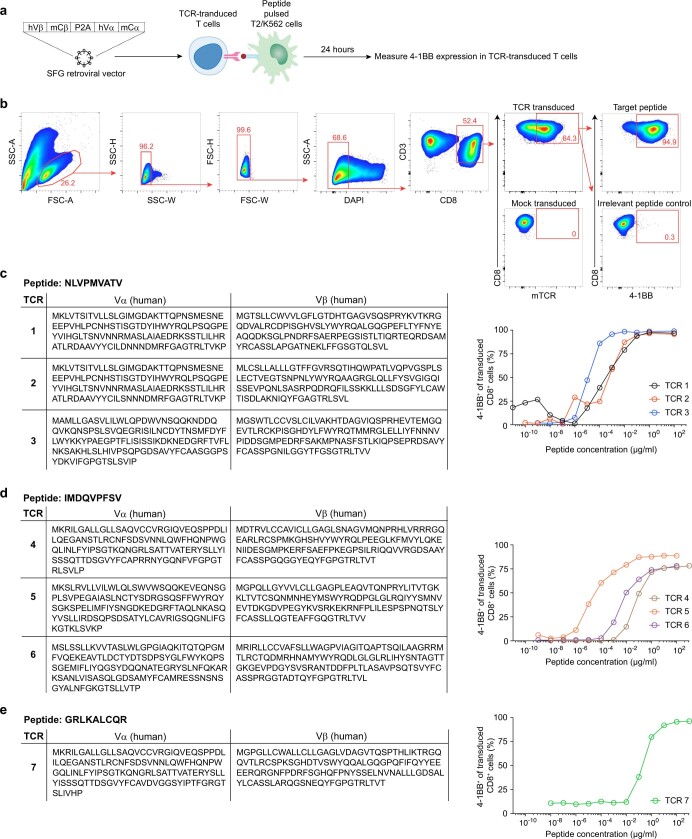
TCR transduction and antigen specificity. (**a**) Experimental schema to transduce and measure ${{\bf{p}}}^{\text{WT}}$-specific T cell receptor (TCR) activation. hVα,β = human α and β variable regions; mCα,β = mouse α and β constant regions. (**b**) Representative gating strategy to detect transduced TCR activation and specificity. (**c**–**e**) Sequences of model ${{\bf{p}}}^{\text{WT}}$s and ${{\bf{p}}}^{\text{WT}}$-specific TCRs, and TCR activation across varied ${{\bf{p}}}^{\text{WT}}$concentrations. Source data

**Extended Data Fig. 5:**
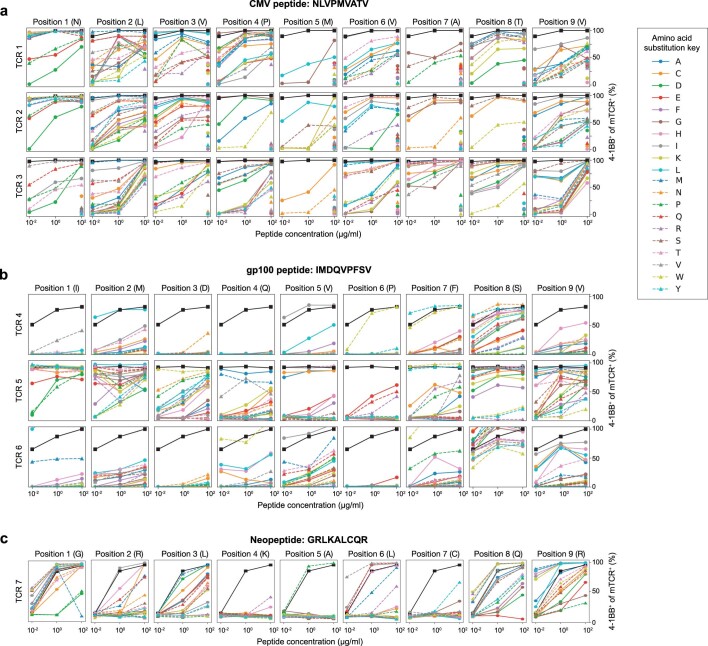
T cell activation is variably degenerate to single amino acid substitutions. (**a**–**c**) T cell activation to model ${{\bf{p}}}^{\text{WT}}$s (black curves) and single amino acid substituted ${{\bf{p}}}^{\text{MT}}$s (color curves). Source data

**Extended Data Fig. 6:**
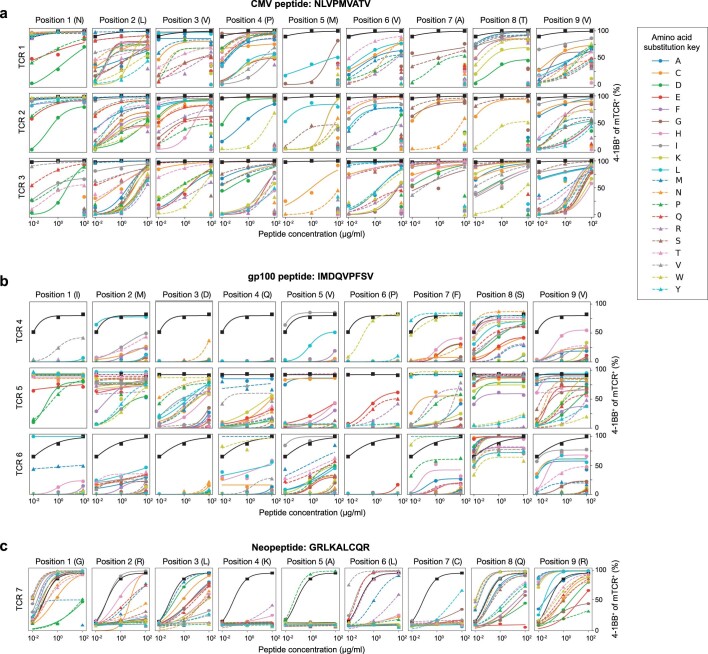
T cell activation to degenerate substitutions follows a sigmoidal function. (**a**–**c**) Fitted T cell activation curves to model ${{\bf{p}}}^{\text{WT}}$s (black curves) and single amino acid substituted ${{\bf{p}}}^{\text{MT}}$s (color curves). Source data

**Extended Data Fig. 7:**
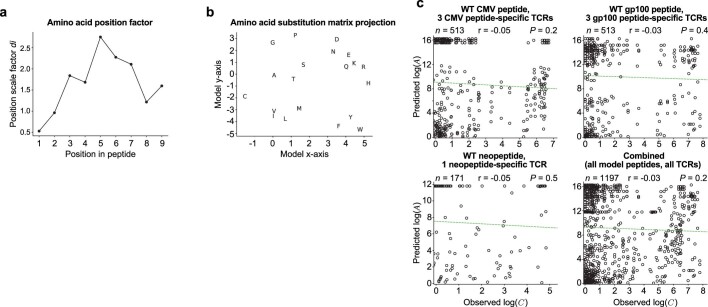
Cross-reactivity distance *C* model. Amino acid position dependent factor (**a**) and substitution matrix (**b**) of cross-reactivity model based on T cell receptor (TCR) cross-reactivity to strong (CMV) and weak (gp100) ${{\bf{p}}}^{\text{WT}}$s and single amino acid substituted ${{\bf{p}}}^{\text{MT}}$s (Fig. 3d, e). (**c**) Correlation of substitution-induced differential MHC-I binding (${\rm{\log }}\left(A\right)$ = ${K}_{{\rm{d}}}^{{\rm{WT}}}/{K}_{{\rm{d}}}^{{\rm{MT}}}$) and substitution induced differential TCR activation (${\rm{\log }}\left(C\right)$ = ${\rm{E}}{{\rm{C}}}_{50}^{{\rm{MT}}}/{\rm{E}}{{\rm{C}}}_{50}^{{\rm{WT}}}$) for all model ${{\bf{p}}}^{\text{WT}}$-TCR pairs and single amino acid substituted ${{\bf{p}}}^{\text{MT}}$s. ${K}_{{\rm{d}}}^{{\rm{WT}}}$ and ${K}_{{\rm{d}}}^{{\rm{WT}}}$ determined through computational predictions of ${{\bf{p}}}^{\text{WT}}$ and ${{\bf{p}}}^{\text{MT}}$ binding to HLA-A*02:01 (CMV, gp100 peptides) and HLA-B*27:05 (tumour neopeptide) with Net MHC 3.4. ${{\rm{EC}}}_{50}^{\text{MT}}$ and ${{\rm{EC}}}_{50}^{{\rm{WT}}}$ measured experimentally through ${{\bf{p}}}^{\text{WT}}$ and ${{\bf{p}}}^{\text{MT}}$ reactivity to TCRs. *n* = individual peptide-TCR measurements. *P* values by two-tailed Pearson correlation (**c**). Source data

To model *C*, we leveraged recent findings that conserved structural features underlie TCR–peptide recognition. Specifically, the binding domains of peptide-degenerate TCRs^21,22^ and TCR-degenerate peptides^23^ share common amino acid motifs, suggesting that T cell cross-reactivity between **p**^MT^ and **p**^WT^ could estimate the relative *C* of different neoantigenic substitutions (Fig. 3b). We selected an HLA-A*02:01-restricted strong epitope (NLVPMVATV (NLV)) from human cytomegalovirus^24^ that was previously used to model TCR–peptide degeneracy^21,22^ as a model **p**^WT^, and three NLV-specific TCRs (Extended Data Fig. 4a–c). We then varied the NLV peptide by every amino acid at each position to model **p**^MT^ substitutions, and compared how TCRs cross-react between each **p**^MT^ and its **p**^WT^ across a 10,000-fold concentration range where **p**^WT^ changes maximally altered T cell activation (Fig. 3b). We observed that substitutions were either highly, moderately or poorly cross-reactive (Fig. 3c, d), and the cross-reactivity pattern depended on the substituted position and residue (Extended Data Fig. 5a). Interestingly, we found similar patterns of cross-reactivity between a model HLA-A*02:01-restricted weaker **p**^WT^ epitope in the melanoma self-antigen gp100^25,26^ (Extended Data Figs. 4d and 5b), three **p**^WT^-specific TCRs and single-amino-acid-substituted **p**^MT^s, suggesting that conserved substitution patterns define *C* (Fig. 3e and Extended Data Fig. 5b). Thus, we quantified the cross-reactivity distance *C* between a **p**^WT^ and its corresponding **p**^MT^ as $\,C\left({{\bf{p}}}^{{\rm{WT}}},{{\bf{p}}}^{{\rm{MT}}}\right)={{\rm{EC}}}_{50}^{{\rm{MT}}}/{{\rm{EC}}}_{50}^{{\rm{WT}}}$. We chose the half maximal effective concentration (EC_50_) to model *C*, as T cell activation to **p**^WT^ was consistently a sigmoidal function (Extended Data Figs. 4c, d and 6a, b) described by a Hill equation, where EC_50_ determines how a ligand activates a receptor. We next estimated the EC_50_ of all 1,026 TCR–**p**^MT^ pairs to infer a model for *C* that estimates whether a neoantigenic substitution is cross-reactive (and therefore tolerated) based on the substituted amino acid position and residue (Extended Data Figs. 6a, b and 7a, b). We then tested whether *C* predicted cross-reactive substitutions in an HLA-B*27:05-restricted neopeptide–TCR pair from an LTS (Extended Data Fig. 4e). Notably, *C* predicted cross-reactive **p**^WT^, **p**^MT^ and **p**^MT^, **p**^MT^ substitutions in this neopeptide–TCR pair (Fig. 3f and Extended Data Fig. 5c, 6c). Thus, we combined all 1,197 TCR–**p**^MT^ pairs to derive a composite *C*—the antigenic distance for a TCR to cross-react between amino-acid-substitution pairs (Fig. 3g and Extended Data Fig. 7c). Broadly, two factors promote cross-reactivity: substitutions at peptide termini^27^ and within amino acid biochemical families (driven by amino acids of similar size and hydrophobicity; Fig. 3g). With this composite *C*, we now define self-discrimination *D* between a **p**^WT^ and its corresponding **p**^MT^ (Fig. 3a) as

1 $$D({{\bf{p}}}^{{\rm{W}}{\rm{T}}}\to {{\bf{p}}}^{{\rm{M}}{\rm{T}}})=(1-w)\log \,\left(\frac{{K}_{{\rm{d}}}^{{\rm{W}}{\rm{T}}}}{{K}_{{\rm{d}}}^{{\rm{M}}{\rm{T}}}}\right)+w\,\log \,\left(\frac{{{\rm{E}}{\rm{C}}}_{50}^{{\rm{M}}{\rm{T}}}}{{{\rm{E}}{\rm{C}}}_{50}^{{\rm{W}}{\rm{T}}}}\right),$$

where $w$ sets the relative weight between the two terms. We chose the parameters of the neoantigen quality model to maximize the log-rank test score of survival analysis on an independent cohort of 58 patients with PDAC^5^ (Supplementary Methods and Extended Data Table 1a).

## Immunoediting of neoantigens

**Extended Data Fig. 8:**
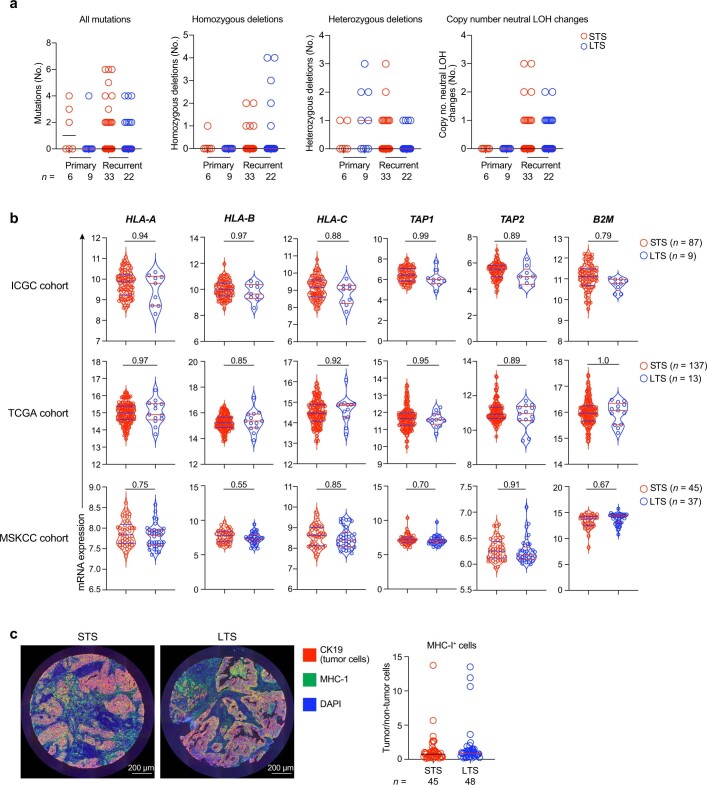
LTS and STS PDACs have equivalent genetic changes in HLA class-I pathway genes. (**a**) Number of mutations (synonymous and non-synonymous), homozygous deletions, heterozygous deletions and copy number neutral loss of heterozygosity (LOH) changes in HLA class-I pathway genes (*B2M*, *CANX*, *CALR*, *HLA-A*, *HLA-B*, *HLA-C*, *HLA-E*, *HLA-F*, *HLA-G*, *TAP1*, *TAP2*, *TAPBP*, *ERAP1*, *ERAP2*, *HSPA5*, *PDIA3*, *SAR1B*, *SEC13*, *SEC23A*, *SEC24A*, *SEC24B*, *SEC24C*, *SEC24D*, *SEC31A*) in primary and recurrent PDACs. (**b**) mRNA expression in HLA class-I pathway genes by bulk RNA sequencing (ICGC, TCGA cohorts) and transcriptional analysis (Affymetrix, Memorial Sloan Kettering Cancer Center (MSKCC) cohort) in primary PDAC tumours. (**c**) Representative multiplexed immunohistochemical images (**left**) and ratio (**right**) of MHC-I^+^ tumour cells (CK19^+^) and MHC-I^+^ non-tumour cells (CK19^-^) in STS and LTS primary PDACs. *n* = individual tumours. Horizontal bars = median. Horizontal bars on violin plots show median and quartiles. *P* value by Wald’s test adjusted for multiple comparison testing. Source data

We applied our model to PDAC, positing that immunoediting will differentially deplete neoantigens with higher *D* (less self) in LTS versus STS PDACs. First, we stratified the frequency of mutations by the antigenic distance as defined by *C* (Fig. 3g and Supplementary Methods). Compared with mutations with a lower antigenic distance, mutations with a greater antigenic distance from self were more significantly depleted in both LTS and STS PDACs (Fig. 3h (left and middle)) and, interestingly, preferentially more depleted in LTS compared with STS PDACs (Fig. 3h (right)). To further examine these observations, we applied the full *D* model to find that neoantigens with both a higher *C* and *D* were strikingly more depleted in LTS versus STS PDACs (Fig. 3i). Interestingly, genes in the HLA class-I pathway were not differentially mutated, deleted, expressed or localized in STS versus LTS PDACs, indicating that neoantigen depletion was not accompanied by acquired resistance in the HLA class-I pathway in LTSs (Extended Data Fig. 8a–c). Thus, tumours in LTSs selectively lose high-quality neoantigens.

## Predicting recurrent tumour composition

**Fig. 4:**
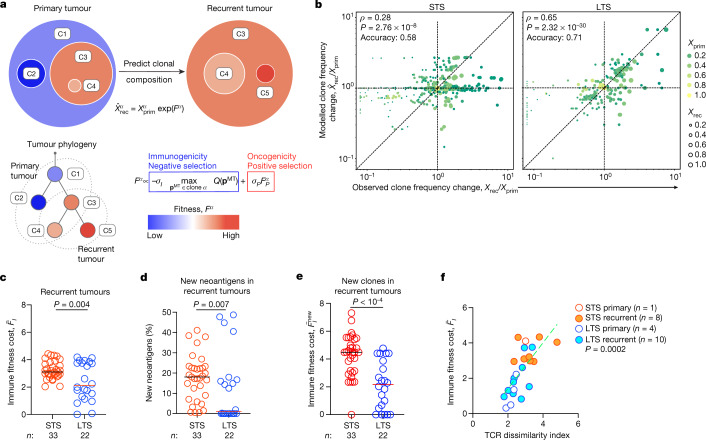
The neoantigen quality fitness model identifies edited clones to predict the clonal composition of recurrent tumours. **a**, Recurrent tumour clone composition prediction based on the primary tumour composition and the fitness model. **b**, Model fitted ${\hat{X}}_{{\rm{rec}}}^{\alpha }/{X}_{{\rm{prim}}}^{\alpha }$ and observed ${X}_{{\rm{rec}}}^{\alpha }/{X}_{{\rm{rec}}}^{\alpha }$ clone frequency changes for the STS (left) and LTS (right) cohorts. Frequency ratios below the sampling threshold were evaluated with pseudocounts. **c**–**e**, The immune fitness cost ${\bar{F}}_{I}$ of recurrent tumours (**c**), new clones (**e**), and the percentage of new neoantigens in recurrent tumours (**d**). **f**, TCR dissimilarity index and immune fitness cost ${\bar{F}}_{I}$ in tumours. *n* indicates the number of tumours. The green line is a linear regression fit. The horizontal bars show the median values*. P* values were determined using two-tailed Spearman correlation (**b**), two-tailed Pearson correlation (**f**) and two-tailed Mann–Whitney *U*-tests (**c**–**e**). Source data

We next incorporated neoantigen quality parameters into a fitness model^4,5^ to test whether our model that predicts clonal tumour evolution can identify immunoedited clones. We reconstructed joint multisample phylogenies^28^ for all tumours from each patient to provide a common clonal structure and track clone frequencies between the tumours of the same patient. To describe selective pressures acting on tumour clones, we accounted for positive selection due to cumulative mutations in driver oncogenes. We quantify this effect in a minimal model ${F}_{P}^{\alpha }$, which counts the number of missense mutations in canonical PDAC driver genes (*KRAS*, *TP53*, *CDKN2A* and *SMAD4*) in each clone *α*. The composite fitness model (Fig. 4a) defines fitness function, *F*^*α*^, of clone *α* as the sum of a negative fitness cost due to immune recognition of high-quality neoantigens and positive fitness gain due to the accumulation of mutations in driver oncogenes,

2 $${F}^{\alpha }=-{\sigma }_{I}\mathop{max}\limits_{{{\bf{p}}}^{{\rm{M}}{\rm{T}}}\in \text{clone}\,\alpha }Q({{\bf{p}}}^{{\rm{M}}{\rm{T}}})+{\sigma }_{P}{F}_{P}^{\alpha }$$

with the free parameters *σ*_*I*_ and *σ*_*P*_ setting the amplitude of the fitness components (Supplementary Methods). We use the model to predict the frequencies of clones propagated to recurrent tumours as

3 $${\hat{x}}_{{\rm{rec}}}^{\alpha }=\frac{1}{Z}{x}_{{\rm{prim}}}^{\alpha }\,\exp ({F}^{\alpha }),$$

where ${x}_{{\rm{prim}}}^{\alpha }$ is the frequency of clone *α* in the primary tumour, ${\hat{x}}_{{\rm{rec}}}^{\alpha }$ is its predicted frequency in the recurrent tumour and constant *Z* ensures correct normalization. We evaluated how closely the fitness model predicted clonal evolution in the recurrent tumours. To do this, for each recurrent tumour in the LTS and STS cohorts, we performed maximum-likelihood fitting of the model parameters *σ*_*I*_ and *σ*_*P*_ in equation (3).

**Extended Data Fig. 9:**
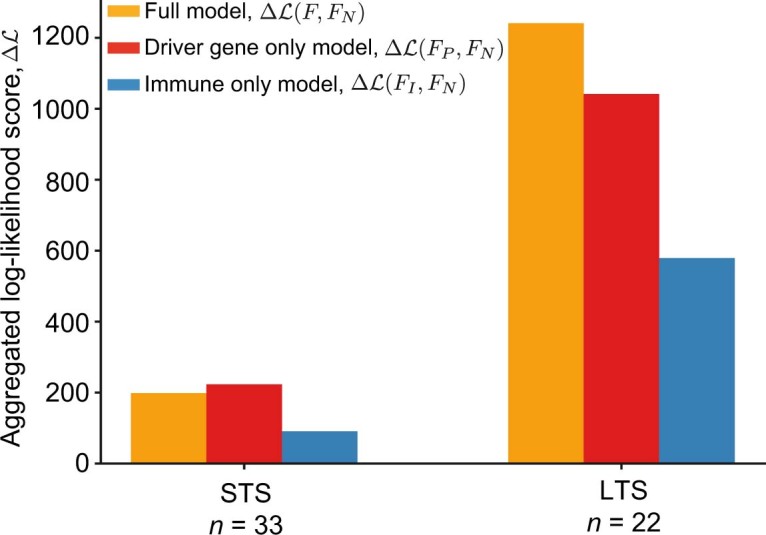
Evaluation of clone fitness model predictions. The log-likelihood score (Supplementary Methods, eq. (31)) is shown for the STS and LTS cohorts to estimate the statistical information gain of fitness models and the amount of evidence of the selective pressures captured by each of the models. The orange bars show the aggregated log-likelihood scores, $\Delta {{\mathscr{L}}}^{\text{STS}}\left(F,{F}_{N}\right)$ and $\Delta {{\mathscr{L}}}^{\text{LTS}}\left(F,{F}_{N}\right),$ of the two-component fitness model, $F$, with parameters ${\sigma }_{I},{\sigma }_{P}$ optimized for each recurrent tumour sample, as compared to the null model, ${F}_{N}$, standing for neutral clone evolution, with zero fitness and parameters ${\sigma }_{I}=0,{\sigma }_{P}=0$. The red bars present the corresponding aggregated log-likelihood scores $\Delta {{\mathscr{L}}}^{\text{STS}}\left({F}_{P},{F}_{N}\right)$ and $\Delta {{\mathscr{L}}}^{\text{LTS}}\left({F}_{P},{F}_{N}\right)$ for the driver-gene only fitness model, ${F}_{P}$, which accounts for positive selection on driver genes but disregards the effect of immune selection, with parameter ${\sigma }_{I}=0,$ and ${\sigma }_{P}$ optimized for each recurrent tumour sample. Finally, the blue bars present the corresponding aggregated log-likelihood scores $\Delta {{\mathscr{L}}}^{\text{STS}}\left({F}_{I},{F}_{N}\right)$ and $\Delta {{\mathscr{L}}}^{\text{LTS}}\left({F}_{I},{F}_{N}\right)$ for the immune-only fitness model, ${F}_{I}$, with parameter ${\sigma }_{P}=0,$ and ${\sigma }_{I}$ optimized for each recurrent tumour sample. Source data

We found that our model provided a better fit of the observed evolution of LTS compared to STS tumour clones, predicting observed evolution in 86% of LTS tumours versus 52% of STS tumours (Extended Data Table 1b) when compared with a neutral model (no selection pressure on clones; differences were quantified with a Bayesian information criterion; Supplementary Methods). Notably, a partial fitness model that incorporates only the oncogenicity component, ${F}^{\alpha }={\sigma }_{P}{F}_{P}^{\alpha }$, showed reduced performance for the LTS tumours but not STS tumours (Extended Data Table 1b and Extended Data Fig. 9). To illustrate this further, we compared observed and model-fitted clone frequency changes between the primary and recurrent tumours, ${X}_{{\rm{rec}}}^{\alpha }/{X}_{{\rm{prim}}}^{\alpha }$ and ${\hat{X}}_{{\rm{rec}}}^{\alpha }/{X}_{{\rm{prim}}}^{\alpha }$ (Fig. 4b), for all reliably predictable clones in the primary tumour (above 3% frequency; Supplementary Methods). The direction of frequency changes was correctly predicted for 71% of LTS and 58% of STS tumour clones (rank correlation *ρ* of 0.65 and 0.28, respectively; Fig. 4b and Extended Data Table 1b). We attribute the model’s better predictions in LTS tumours to the presence of immune selection in these tumours.

Next, we computed the overall tumour immune cost (averaging the immune component, ${F}_{I}^{\alpha }=\mathop{max}\limits_{{{\bf{p}}}^{{\rm{MT}}}\in \mathrm{clone}\alpha }Q({{\bf{p}}}^{{\rm{M}}{\rm{T}}})$ over all tumour clones). Consistently, the immune fitness cost was lower in recurrent LTS tumours compared with in STS tumours (Fig. 4c). Furthermore, we considered the immune cost only of clones that are new in recurrent tumours, but not present in primary tumours. Recurrent LTS tumours contained both fewer new neoantigens (1% versus 18%; Fig. 4d) and new clones with markedly lower immune fitness cost (Fig. 4e) compared with recurrent STS tumours. These observations again suggest that the LTS recurrent tumours had been subject to immunoediting.

Finally, we confirmed these results by analysing TCR sequencing data in the available recurrent tumour samples. We quantified the specificity of T cell clonal expansion using the TCR dissimilarity index^18^ (Supplementary Methods and Extended Data Fig. 1a, b) and correlated this index to immune fitness cost. We found greater T cell clonal expansion in tumours (lower TCR dissimilarity index) correlated with more highly edited tumours (lower immune fitness cost) (Fig. 4f and Extended Data Fig. 1c). In summary, these results strongly suggest that neoantigens are immunoedited in PDAC, and that our fitness model captures the selective pressures by T cells acting on tumour clones.

## Discussion

Here we clarify several questions on how the immune system interacts with cancer. First, does cancer immunoediting occur in humans? As the theory of cancer immunoediting was developed by studying carcinogen-induced highly mutated murine sarcomas^1,3^, it has remained uncertain whether these principles apply to human cancers^29–31^. We postulated that spontaneous immunoediting of a human cancer should manifest when the immune system recognizes an immunogenic antigen in a primary tumour, as this should induce the antigen to be subsequently eliminated in the recurrent tumour. Indeed, this is what we found—tumours that evolve under stronger immune pressure lose more immunogenic neoantigens. Although we did not assess the changes in non-mutated antigens or address how different cellular compositions and tissue environments may modulate editing, it is notable that the proof for immunoediting is revealed in PDAC, a low-mutated cancer that is considered to be resistant to endogenous immunity. This strengthens the claim that immunoediting is a broadly conserved principle of carcinogenesis.

Second, does immunoediting manifest as loss of immunogenic antigens, or do cancers also acquire genetic resistance? Interestingly, we observed the former but not the latter. We postulate that such phenotypes are governed by the magnitude of the selective pressure. Although LTSs exhibit higher immune pressures in tumours than STSs, this is ostensibly still lower than pharmacologically boosted immune pressure in a tumour^32^. Thus, in LTSs, as pressure is moderate, tumours lose immunogenic antigens; by contrast, where pressure is maximal, such as perhaps when under therapy, tumours acquire resistance^32^. This distils cancer evolution under immune selection to a simpler concept—selection determines clonal composition, and pressure determines adaptive change. Further studies will test these concepts.

Third, can we quantify how the immune system recognizes mutations?  We combined experimental techniques and machine learning to present a new metric that captures how T cells cross-react between peptides. We use *C* to quantify the antigenic distance of mutated peptides in the TCR-recognition space and the qualities that render individual mutations immunogenic, building on our previous efforts^4,5^ to formalize antigen quality. Although we used our quality model to identify immunogenic neoantigens, we propose that it captures common immunogenic features in antigens. Thus, we anticipate that our model can further illuminate the biology of antigens beyond cancer, including T cell cross-reactivity between antigens, pathologies of cross-reactivity (such as autoimmunity) and therapies that require rational antigen selection (such as vaccines).

Finally, it is notable that quantifying the ability of the immune system to discriminate changes in mere single amino acids can predict how cancers evolve. This undoubtedly reflects that a fundamental function of the immune system is to maintain integrity of the host genome. We therefore speculate that our model in essence captures the mechanisms through which the immune system preserves genomic integrity.

### Reporting summary

Further information on research design is available in the Nature Research Reporting Summary linked to this paper.

## Online content

Any methods, additional references, Nature Research reporting summaries, source data, extended data, supplementary information, acknowledgements, peer review information; details of author contributions and competing interests; and statements of data and code availability are available at 10.1038/s41586-022-04735-9.

### Supplementary information

Supplementary Information Supplementary Table 1, legends for Supplementary Tables 2 and 3, and Supplementary Methods.

Reporting Summary

Supplementary Table 2

Supplementary Table 3

### Source data

Source Data Fig. 1

Source Data Fig. 2

Source Data Fig. 3

Source Data Fig. 4

Source Data Extended Data Fig. 1

Source Data Extended Data Fig. 2

Source Data Extended Data Fig. 4

Source Data Extended Data Fig. 5

Source Data Extended Data Fig. 6

Source Data Extended Data Fig. 7

Source Data Extended Data Fig. 8

Source Data Extended Data Fig. 9

## Figures and Tables

**Extended Data Table 1:**
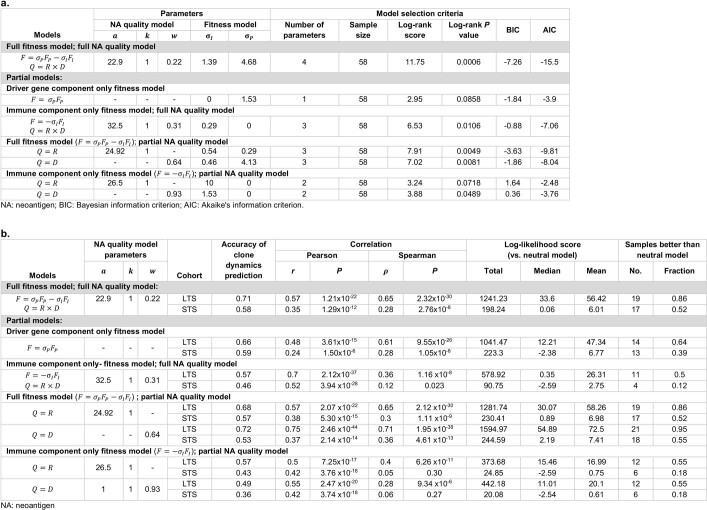
Neoantigen quality fitness models

Full and partial neoantigen quality fitness models to predict survival (a) and recurrent tumour clone composition (b). We provide additional details on the respective models in the

## Data Availability

All raw sequencing data obtained through the Johns Hopkins Hospital medical donation programme have been previously described^19^ and are available at the European Genome–Phenome Archive under accession number EGAS00001004097. All other raw sequencing data are available at the NCBI Sequence Read Archive under accession number PRJNA648923. The ICGC data used in this study are available at the ICGC (https://dcc.icgc.org/repositories) under the identifier PACA-AU. The TCGA data used in this study are from TCGA-PAAD dataset available at the NCI Genomic Data Commons (https://gdc.cancer.gov/). Source data are provided with this paper.

## Code availability

Code used to construct and apply the model is available at GitHub (https://github.com/LukszaLab/NeoantigenEditing).
