# Supplementary material for: Neoantigen quality predicts immunoediting in survivors of pancreatic cancer
Source: Nature. 2022 May 19;606(7913):389–95. doi: 10.1038/s41586-022-04735-9 (PMC9177421; doi:10.1038/s41586-022-04735-9)
Supplement: Supplementary file 1 — Supplementary Table 1, legends for Supplementary Tables 2 and 3, and Supplementary Methods. [file 41586_2022_4735_MOESM1_ESM.pdf]

nature portfolio

https://doi.org/10.1038/s41586-022-04735-9

Supplementary information

Neoantigen quality predicts immunoediting in survivors of pancreatic cancer

In the format provided by the authors and unedited

Supplementary Information

Neoantigen quality predicts immunoediting in survivors of pancreatic cancer

Łuksza, M et al.

Table of contents

Supplementary Table 1……………………………………………………………………......................2

Supplementary Table 2 description…………………………………………………………………..3

Supplementary Table 3 description…………………………………………………………………..3

Methods…………………………………………………………………………………………4

Supplementary Table 1: Clinicopathologic characteristics of the matched primary and recurrent PDAC cohort **(Figure 1a).**

Characteristic	Short-term survivors (STSs) (*n* = 6) *n* (%)	Long-term survivors (LTSs) (*n* = 9) *n* (%)	*P* value*
Sex			
Male	3 (50)	4 (44)	
Female	3 (50)	5 (56)	0.83
Age (y)			
Median (range)	63 (54-74)	60 (44-71)	0.37
Tumor Location			
Head	6 (100)	6 (67)	
Body/tail	0 (0)	3 (33)	0.11
Surgery for primary tumor			
Distal pancreatectomy	0 (0)	3 (33)	0.11
Pancreaticoduodenectomy	6 (100)	6 (67)	
Pathological stage at diagnosis			
I	0 (0)	3 (33)	
II	5 (83)	5 (56)	
III	1 (17)	1 (11)	
IV	0 (0)	0 (0)	0.28
Margin			
Positive	1 (17)	3 (33)	
Negative	5 (83)	6 (67)	0.71
Adjuvant chemotherapy			
Yes	6 (100)	8 (89)	
No	0 (0)	1 (11)	0.39
Chemotherapy on recurrence			
Yes	5 (83)	6 (67)	0.47
No	1 (16)	3 (33)	
Metastasectomy	0 (0)	6 (67)	0.0009
Site of metastasectomy			
Lung	NA	4 (44)	
Ovary		2 (22)	

* *P* value by Chi-square test

Supplementary Table 2: This table is provided in Excel format file, and contains a comprehensive list of all neopeptide sequences and HLA alleles with predicted neopeptide binding.

Supplementary Table 3: This table is provided in Excel format file, and contains a comprehensive list of all human infectious derived, class-I restricted peptide sequences with positive immune assays derived from the Immune Epitope Database used in this study.

Methods

Patient Samples

We collected matched primary and recurrent PDACs through surgical resection at Memorial Sloan Kettering Cancer Center (MSK) (n = 5/9 LTS), and the Garvan Institute of Medical Research (n = 1/9 LTS) (**Supplementary Table 1**). Additional matched primary and recurrent PDACs were previously obtained through the Gastrointestinal Cancer Rapid Medical Donation Program at The Johns Hopkins Hospital (JHH) (n = 3/9 LTS, 6/6 STS) and have been previously described[19] (**Supplementary Table 1**) under Institutional Review Board-approved study protocols. Cohorts of primary only PDAC were collected at MSK (MSK primary PDAC cohort), the International Cancer Genome Consortium (ICGC primary PDAC cohort), and The Cancer Genome Atlas (TCGA) through surgical resection as previously described.[5,33] We obtained informed consent from all patients. We performed the study in strict compliance with all institutional ethical regulations and institutional review boards. All tumor samples were PDACs. We excluded adenocarcinomas in cystic pancreatic neoplasms and neuroendocrine tumors. We defined LTS and STS consistent with our previous work.[5] We identified all tumors through histopathologic evaluation following surgery or at autopsy. We conservatively estimated that patients had 100 recurrent tumors when the number of tumors were too numerous to count.

Nucleic acid extraction, whole exome sequencing and mutation identification

We previously described methods to extract DNA and sequence samples collected at MSK, Garvan Medical Center,[5] and JHH.[19] All samples from MSK, Garvan, and JHH were examined by an expert GI pathologist and confirmed to have at least 20% neoplastic cellularity and preserved tissue quality. We macrodissected samples meeting these criteria from serial unstained sections, and extracted genomic DNA as previously described.[5,19] 500 ng of genomic DNA was then fragmented to a target size of 150-200 bp on a LE220 ultrasonicator (Covaris). Barcoded libraries (Kapa Biosystems) were subjected to exon capture by hybridization using the SureSelect Human All Exon 51MB V3 (JHH samples) or V4 (all other samples) kits (Agilent). DNA libraries were subsequently sequenced on a HiSeq 2500 (JHH samples) or 4000 (all other samples) (Illumina) in paired end 100/100 reads, using the TruSeq SBS Kit v3 (Illumina) with target coverage of 150-250X for tumor samples and 70X for matched normal. Sequence data were demultiplexed using Illumina CASAVA software. After removal of adapter sequences using BIC,[34] reads were aligned to the reference human genome (hg19) using the Burrows-Wheeler Alignment tool (bwa mem v0.7.17) and samtools (v1.6). Duplicates were marked with picard-2.11.0 MarkDuplicates (http://broadinstitute.github.io/picard). Indel realignments were done with the Genome Analysis toolkit (GenomeAnalysisTK-3.8-1-0-gf15c1c3ef) RealignerTargetCreator and IndelRealigner[35] using 1000 genome phase1 indel (1000G_phase1.indels.b37.vcf) and Mills indel calls (Mills_and_1000G_gold_standard.indels.b37.vcf) as references. Base calls were recalibrated with BaseRecalibrator[35] and dbSNP version 138. Average unique sequence coverages of 207X, 152X, 212X, and 221X were achieved for STS primary, LTS primary, STS recurrent, and LTS recurrent tumor samples respectively, and 88X and 84X were achieved for STS normal and LTS normal samples, respectively.

MuTect 1.1.7[35] and Strelka 1.0.15[36] were used to call SNVs and indels on pre-processed sequencing data. For the MuTect calls, dbSNP 138 and CosmicCodingMuts.vcf version 86[37] were used as reference files. For the Strelka calls, we set "isSkipDepthFilters = 1" to prevent filtering-out of mutation calls from exome sequencing due to exome-sequencing mapping breadth. Unbiased normal/tumor read counts for each SNV and indel call were then assigned with the bam-readcount software 0.8.0 (https://github.com/genome/bam-readcount). A minimum base quality filter was set with the "-b 15" flag. The reads were counted in an insertion-centric way with the "-i" flag, so that reads overlapping with insertions were not included in the per-base read counts. We then use the normal/tumor read counts to filter mutations. Filtering criteria are 1) total coverage for tumor $\geq$10, 2) variant allele frequency (VAF) for tumor $\geq$4%, 3) number of reads with alternative allele $\geq$ 9 for tumor, 4) total coverage for normal $\geq$7, and 5) VAF for normal $\leq$1% at a given mutation. These filters exist for all mutations except for mutations in the KRAS gene.

In order to avoid missing possible *KRAS* driver mutations, common mutations in *KRAS* known to be pathogenic were manually curated if the sample did not already contain a *KRAS* mutation. Using IGV Viewer 2.4.19,[38] we inspected the top ten coding mutations in *KRAS* denoted in the Genomic Data Commons Database[39] at the following positions on chromosome 12; bases 25378562(C$>$T), 25380275(T$>$G), 25398281(C$>$T), 25398282(C$>$A), 25398284(C$>$A, T, or G), and 25398285(C$>$A, T, or G). One mutation was selected at most

based on the number of reads containing the alternative allele at the position. In total, six additional *KRAS* mutations were added for the STS samples, and six were added to the LTS samples.

HLA typing

HLA-I typing for PDAC patients was performed in silico with the OptiType version 1.3.3 tool[40] using non-tumor sequencing reads.

Neoantigen prediction

Putative neoantigens were identified in silico. In brief, all wild-type (WT) and mutant genomic sequences corresponding to coding mutations were translated to an amino acid sequence consistent with the GRCh37 reference genome (GRCh37.75) using the snpEff.v4.3t software[41] with options set as "-noStats -strict -hgvs1LetterAa -hgvs -canon -fastaProt [fasta file name]". Only annotations without "WARNING" or "ERROR" were kept and the most deleterious missense mutation was prioritized in mapping a genomic mutation to a gene. Missense mutations were centrally located in a peptide of up to 17 amino acids long, which depended on the location of the missense mutation within a protein. This corresponded to nine 9-mers in a left-to-right sliding fashion, each containing the mutant amino acid in a different position. Predictions of MHC class-I binding for both the WT peptide ($\mathbf{p}^{WT}$) and mutant peptide ($\mathbf{p}^{MT}$) were estimated using the NetMHC 3.4 software[42,43] with patient-specific HLA-I types. All $\mathbf{p}^{MT}$s with predicted IC_{50} affinities below 500 nM to a patient-specific HLA-I type were defined as neoantigens.

Cell lines and cell culture

We purified peripheral blood mononuclear cells (PBMCs) from healthy donor buffy coats (New York Blood Center, New York, USA) and isolated T cells using a Pan-T cell isolation kit (Miltenyi). We activated T cells with CD3/CD28 beads (Thermo Fisher, MA, USA) with IL-7 (3000 IU/mL) and IL-15 (100 IU/mL) (Miltenyi Biotec, Germany), and transduced T cells on day 2 post activation. Virus-producing cell lines (H29 and RD114-envelope producers) were previously described.[44,45] We cultured T cells or HLA-transduced K562 cells and T2 cells in RPMI media supplemented with 10% fetal bovine serum (FBS, Nucleus Biologics), 100 U/ml Penicillin/Streptomycin (Gibco), and 2 mM Glutamine (Gibco). We cultured virus-producing cell lines in DMEM media supplemented with 10% FBS (Nucleus Biologics), 100 U/ml Penicillin/Streptomycin (Gibco), and 2 mM Glutamine (Gibco).

TCR cloning, transduction, and peptide stimulation

We constructed TCR fragments as previously described.[46] Briefly, we fused epitope specific TRB V-D-J and TRA V-J sequences to mouse constant TRB and TRA chain sequences respectively (kind gift of Alena Gros), to prevent mispairing of transduced TCRs with the endogenous TCRs.[47] We used modified mouse constant regions to further improve pairing and increase cell surface TCR expression as previously described (mTCR).[46] We joined the TRB and TRA chains with a furin SGSG P2A linker, cloned the TCR constructs into an SFG γ-retroviral vector,[48] and sequence-verified all plasmids (Genewiz). We transfected retrovirus vectors into H29 cells (gpg29 fibroblasts) using calcium phosphate to produce VSV-G pseudo-typed retroviruses.[44] We next used Polybrene (Sigma) and viral-containing supernatants to generate stable RD114-enveloped producer cell lines.[45] We collected and concentrated virus-containing supernatants using Retro-X™ Concentrator (Takara). For T cell transductions, we coated non-tissue culture treated 6-well plates with Retronectin (Takara) as per the manufacturer's protocol. We plated a titrated viral quantity to 3×10^6 activated T cells per well, and centrifuged cells for 1 hour at room temperature at 300g, and used transduced T cells either between day 7-14 post transduction or cryopreserved them for future use. We used mock-transduced T cells as controls.

To stimulate transduced T cells with peptide, we used T2 cells as antigen presenting cells (APCs). All peptide panels were commercially custom synthesized by Genscript (Piscataway, NJ), and were >95% pure. Briefly, we pulsed 5×10^4 T2 cells per well in a 96-well U-bottom plate for 1 hour at 37°C with the indicated peptide at the indicated concentrations. After 1 hour, we washed out the peptide by centrifugation, and added 5×10^4 TCR-transduced T cells per well. We used an equivalent number of mock transduced total T cells, and irrelevant peptides as controls. We measured CD137 (4-1BB) expression on $CD8^+$ $mTCR^+$ T cells 24 hours later.

Flow cytometry

We defined TCR transduced $CD8^+$ T cells as live, $CD3^+$, $CD8^+$, $mTCR^+$ cells (**Extended Data Figure 4b**). We purchased antibodies from Biolegend (CD3 - clone SK-7, PE-Cy7; CD8 - clone SK1, Alexa Fluor 700; mTRB - clone H57-597, PE-Cy5; and CD137 - clone 4B4-1, PE), and viability dye (DAPI solution) from BD Biosciences.

We stained cells using antibody cocktails in the dark at 4°C according to manufacturer's instructions, washed, and analyzed on a FACS LSR Fortessa (BD Biosciences). Flow cytometric data were collected using FACSDiva (BD Biosciences, version 8.0.1). We diluted reagents according to manufacturer's instructions. We used Flowjo (version 10, Tree Star) to perform our analyses.

Neoantigen quality model: Q

We consider 9-amino-acid long peptides containing a single point mutation as potential neoantigens if they are a predicted binder to a patient specific HLA allele. For a given neoantigen with a peptide sequence $\mathbf{p}^{\mathrm{MT}}$ (with corresponding wildtype peptide sequence denoted as $\mathbf{p}^{\mathrm{WT}}$), and an HLA allele h, we define the neoantigen quality Q as:

$$Q(\mathbf{p}^{\mathrm{MT}}, h) = R(\mathbf{p}^{\mathrm{MT}}) \times D(\mathbf{p}^{\mathrm{MT}}, \mathbf{p}^{\mathrm{WT}}, h)\ . \quad (1)$$

The non-self recognition component R quantifies the recognition probability of peptide $\mathbf{p}^{\mathrm{MT}}$ by comparison to validated non-self epitopes from infectious diseases and it has been introduced previously.[4,5] The self discrimination component, D, expands on the previously proposed measure based only on MHC-presentation[4,5] by including a new measure of cross-reactivity distance, C, between $\mathbf{p}^{\mathrm{MT}}$ and $\mathbf{p}^{\mathrm{WT}}$. Below we briefly describe R and derive D.

Non-selfness: R

To estimate the "non-selfness" of a peptide we calculate the similarity of a given peptide to epitopes which have been previously recognized as non-self. To do so we estimate a recognition probability R of a peptide with sequence **p** based on its sequence similarity to a dataset of recognizable epitopes **e**. Here similarity is measured using a gapless alignment with BLOSUM62, though this can be easily generalized. We use a thermodynamically motivated model,[4,5]

$$R(\mathbf{p}) \equiv R(\mathbf{p}, a, k) = Z(\mathbf{p}, a, k)^{-1} \sum_{\mathbf{e}} \exp\left(-k(a - |\mathbf{p}, \mathbf{e}|)\right), \quad (2)$$

where $Z(\mathbf{p}, a, k) = 1 + \sum_{\mathbf{e}} \exp\left(-k(a - |\mathbf{p}, \mathbf{e}|)\right)$ is the normalization constant, $|\mathbf{p}, \mathbf{e}|$ is a local alignment score between **p** and **e**, and free parameters a and k represent the horizontal displacement of the binding curve and the slope of the curve. In our case, recognizable epitopes come from the Immune Epitope Database (IEDB),[49] and we restrict our search to all human infectious disease class-I restricted targets with positive immune assays. As the peptides in IEDB can change over time, we use the current version of IEDB and list the positive epitopes used (**Supplementary Table 3**). The parameters of the model are set to optimize the separation of survival curves (detailed description of parameter training is included in later sections). To find the set of IEDB epitope sequences with sequence similarity to neoantigens in our cohort, we used the blastp algorithm with the BLOSUM62 matrix (gap opening penalty=-11, gap extention penalty=-1). We calculated alignment scores with the Biopython Bio.pairwise2 package (http://biopython.org) for all alignments identified with blastp.

Cross-reactivity distance: C

To model a cross-reactivity distance we measure and analyze TCR-pMHC avidity curves, by which we mean activation of a monoclonal T cell population as a function of pulsed exogenous peptide concentration. We define the cross-reactivity distance C between the two peptides as ratio of the EC_{50} of the two avidity curves to a T cell clone that is specific to at least one of the peptides. If the EC_{50} shift between the two avidity curves is small this reflects that the TCR is specific and highly cross-reactive to both peptides; consequently the peptides have a low cross-reactivity distance C. The reverse, a large shift in the EC_{50}, indicates a lack in reactivity against one of the peptides, low cross-reactivity, and thus a $large$ cross-reactivity distance C. Formally, this quantity could depend on the TCR and the HLA allele, however we fit a minimal model for peptides that are one amino acid substitution from each other with the intention of extracting coarse grain features that are sufficiently robust for our application.

Fitting avidity curves

To fit our model we measured avidity curves (**Figure 3c**, **Extended Data Figure 4c-e**) corresponding to seven different peptide-TCR combinations: 3 TCRs specific to a CMV epitope (NLVPMVATV), 3 TCRs specific to a gp100 epitope (IMDQVPFSV), and 1 TCR specific to a neoantigen arising from a mutation in the RHBDF2 gene (GRLKALCQR). For each peptide-TCR combination we measured the avidity curves for the wildtype peptide along with all 171 peptides one amino acid substitution away from the wildtype peptide (1204 total TCR-pMHC combinations)(**Extended Data Figure 5a-c**). For each peptide we extract the EC_{50} from the TCR-pMHC reactivity

curve by fitting a generalized Hill function:

$$V(c) = \frac{V_\infty}{1 + \left(\frac{\mathsf{EC}_{50}}{c}\right)^n} . \tag{3}$$

This function has 3 parameters: the maximum amplitude V_∞, the cooperativity n, and, the term to be inferred, the EC_{50}. As we have 3 concentration points for each peptide, regularization is key to a robust fit of these curves. To motivate the regularization, we use the priors that $V_\infty \approx 1$ (i.e. at infinite peptide concentration, TCR reactivity approaches 100%) and $n \approx 1$ (cooperativity of 1 is for the case of simple binding reactions of 2 molecules). Finally, we enforce a slight regularization on the EC_{50} if it extends outside of the measured concentration region. We use an L2 cost and regularization to fit, yielding a cost function to minimize:

$$\sum_c (V(c) - V_{\mathsf{meas}}(c))^2 + r_V (1 - V_\infty)^2 + r_n (n-1)^2 + r_{\mathsf{EC}_{50}} d(\mathsf{EC}_{50}, [0.01, 100])^2. \tag{4}$$

Where $d(\mathsf{EC}_{50}, [0.01, 100])$ indicates the log distance to the measured concentration range of [0.01μg/mL,100μg/mL] (i.e. $d(\mathsf{EC}_{50}, [0.01, 100]) = \max(0, \log(\mathsf{EC}_{50}) - \log(100), \log(0.01) - \log(\mathsf{EC}_{50}))$. The regularization constants used were $r_V = 0.01$, $r_n = 0.01$, and $r_{\mathsf{EC}_{50}} = 0.001$. Parameters were then fit using standard least squares. The inferred EC_{50}'s were further clipped to the range of 10^{-4} μg/mL to 10^4 μg/mL.

Cross-reactivity distance model

To model the effect of a single amino acid substitution on an avidity curve's EC_{50}, we assume that there is a position independent amino acid substitution matrix $\mathcal{M}$ that is rescaled by a position dependent factor d_i. Together this yields a model of the following form for the cross-reactivity distance C between two peptides, $\mathbf{p}^{\mathsf{A}}$ and $\mathbf{p}^{\mathsf{B}}$, which differ only by a single mismatched amino acid in position i:

$$\left|\log\left(C(\mathbf{p}^{\mathsf{A}}, \mathbf{p}^{\mathsf{B}})\right)\right| = \left|\log\left(\frac{\mathsf{EC}_{50}(\mathbf{p}^{\mathsf{B}})}{\mathsf{EC}_{50}(\mathbf{p}^{\mathsf{A}})}\right)\right| = d_i \mathcal{M}(p_i^{\mathsf{A}}, p_i^{\mathsf{B}}). \tag{5}$$

This form of the model for C has more parameters than can be reliably be inferred from our experimentally measured TCR-pMHC avidity curves - the distance weight d_i has 9 parameters, and the substitution matrix $\mathcal{M}$ has 380 free parameters (190 if we assume a symmetric matrix).

To ameliorate this problem, we implement two modifications to reduce the effective number of parameters - first, we embed the 20 amino acids into a bounded 2D region (a 20×20 square) and define the values of the substitution matrix $\mathcal{M}$ as the Euclidean distance between the positions of each embedded amino acid. This reduces the number of free parameters for $\mathcal{M}$ from 190 to 40 and allows for clear visualization of amino acid clustering.

Second, we introduce the BLOSUM62 substitution matrix as a prior (we find a model inference performed without this assumption shows that the inferred substitution matrix correlates significantly to BLOSUM62). We define a cost function that includes not only the differences between the measured and modeled distances but also a regularization term that reflects how well a linear transformation of the BLOSUM62 matrix matches the inferred substitution matrix (we exclude the diagonal terms from this fit as those terms are not fit under the model). The full expression is:

$$\frac{1}{\left|\{\mathbf{p}^{\mathsf{A}}, \mathbf{p}^{\mathsf{B}}\}\right|} \sum_{\{\mathbf{p}^{\mathsf{A}}, \mathbf{p}^{\mathsf{B}}\}} \left(\left|\log\left(C_{\mathsf{model}}(\mathbf{p}^{\mathsf{A}}, \mathbf{p}^{\mathsf{B}})\right)\right| - \left|\log\left(C_{\mathsf{meas}}(\mathbf{p}^{\mathsf{A}}, \mathbf{p}^{\mathsf{B}})\right)\right|\right)^2 + r_{\mathsf{bl62}} \mathsf{RSS}_{\mathsf{bl62}} , \tag{6}$$

where we sum over pairs of measured peptides $\{\mathbf{p}^{\mathsf{A}}, \mathbf{p}^{\mathsf{B}}\}$ and $\mathsf{RSS}_{\mathsf{bl62}}$ is the sum of the square residuals of the optimal linear regression between $\mathcal{M}$ and BLOSUM62.

We used a value of $r_{\mathsf{bl62}} = 0.01$ for the constant that controls the relative weighting of the fit to the measured data or the fit to BLOSUM62. We then use the dual annealing method to minimize the cost function and fit the model parameters.

This model is inferred using the measured log distance between the EC_{50} of two peptides to the same TCR. We restrict the peptide pairs we consider in our inference by requiring both that the peptides are a single amino acid

substitution away from each other and that there is some minimal reactivity to at least one of the peptides to the associated TCR. We set this reactivity threshold as the criteria that the EC_{50} of at least one of the peptides must be less than 0.1μg/mL. This criteria will include all pairs that include one of the wildtype peptides (NLVPMVATV, IMDQVPFSV, or GRLKALCQR), but may also include pairs of mutants that have substitutions in the same position in the wildtype (e.g. NLV**M**MVATV and NLV**K**MVATV). Including these additional combinations allows us to more accurately resolve amino acid substitutions not observed from the original 3 wildtype peptides.

Cross-reactivity model validation

To validate the cross reactivity model C, we inferred a model using peptide pairs only from the NLV and gp100 TCRs (6 TCRs in total) (**Extended Data Figure 7a, b**) and predicted on the remaining RHBDF2 neoantigen peptide pairs (with the same minimum reactivity restrictions as described above for the inference). The NLV and gp100 peptides are presented on HLA-A02:01 whereas the RHBDF2 neoantigen is predicted to be presented on HLA-B27:05, so the validation dataset stems from not only a different wildtype peptide-TCR combination, but also a wholly different HLA allele. We find that the model learned on the NLV and gp100 TCRs provides highly significant predictive power for the peptide pairs from the RHBDF2 neoantigen (**Figure 3f**).

Self discrimination: D

The self discrimination component quantifies how easily $\mathbf{p}^{\text{MT}}$ and $\mathbf{p}^{\text{WT}}$ can be distinguished from each other as a result of negative selection, and is a sum of terms relating to both the MHC presentation and our experimentally derived cross-reactivity distance C. For a given HLA allele h, we calculate the peptide-MHC-I dissociation constants,[50] $K_d^{\text{MT}} \equiv K_d(\mathbf{p}^{\text{MT}}, h)$ and $K_d^{\text{WT}} \equiv K_d(\mathbf{p}^{\text{WT}}, h)$ for both peptides. We consider the relative MHC dissociation constants between $\mathbf{p}^{\text{MT}}$ and its $\mathbf{p}^{\text{WT}}$ counterpart, as the ratio of their inferred MHC-I binding affinities.

We define the combined self discrimination D of a neoantigen as:

$$D(\mathbf{p}^{\text{MT}}, \mathbf{p}^{\text{WT}}, h) = (1-w)\log\frac{K_d(\mathbf{p}^{\text{WT}}, h)}{K_d(\mathbf{p}^{\text{MT}}, h)} + w\log\frac{\text{EC}_{50}(\mathbf{p}^{\text{MT}})}{\text{EC}_{50}(\mathbf{p}^{\text{WT}})}\ . \tag{7}$$

Each term represents an affinity difference, or discrimination energy, between $\mathbf{p}^{\text{MT}}$ and $\mathbf{p}^{\text{WT}}$ either for MHC presentation or for T cell activation. The self discrimination D therefore increases if either the underlying mutation leads to an increased presentation probability, or if it results in a peptide not cross-reactive with the wildtype and thus recognized by a collection of TCRs distinct from those that recognize the wildtype peptide. Parameter $w \in [0, 1]$, sets the relative weight between the two terms: MHC presentation and T cell activation.

Amino acid clustering and subsequent ordering

The dendogram and the amino acid ordering in **Figure 3g** were computed by unsupervised agglomerative clustering using the sklearn package with 0 distance thresholding (sklearn.cluster.AgglomerativeClustering). The distances used for the clustering were the Euclidean distances arising from the 2D embedding of the amino acids in **Figure 3g**.

Mutation and neoantigen distributions

The substitution frequency scatter plots (**Figure 3h**) are generated by determining all nonsynonymous mutations. We determine the corresponding amino acid substitution frequencies by binning the mutation substitutions (e.g. leucine to isoleucine, L$\rightarrow$I) for each nonsynonymous mutation and then normalizing by the total number. Each substitution has a particular score from our inferred amino acid substitution matrix $\mathcal{M}$, which we use as the x-axis. The linear fits are done using least squares regression and Pearson correlations are computed. Unseen amino acid substitutions (generally arising from requiring at least 2 nucleotide mutations) are excluded from the analysis.

The cumulative probability distributions (**Figure 3i**) are computed by determining the total fraction of neoantigens in the defined cohort that have a C or D larger than and or equal to the value on the x-axis.

Clonal structure of tumors

Tumor clones are reconstructed using the PhyloWGS algorithm.[28] We use multisample reconstruction, combining all primary and recurrent samples from a given patient. The algorithm returns a family of 10000 trees, each associated with a likelihood, $(\mathcal{T}_i, L_i)$. When appropriate, our tree-based statistics for a tumor sample will be reported as averages over the top scoring trees, with weight of the i tree defined as $w_i = L_i / \sum_{j=1}^{5} L_j$, the

averaging operator will be denoted as $\langle . \rangle_{\mathcal{T}}$. We empirically checked that the the full set of results are consistent with those that use only the 5 top scoring trees.

A given tree $\mathcal{T}$ provides a common clone topology for all samples in the patient, with clone definitions informed by clustering of mutations across all the samples. For a given clone $\alpha \in \mathcal{T}$, the algorithm estimates the maximum likelihood clone frequency, $X^\alpha \equiv X^\alpha(\mathcal{T})$, which is equivalent to the cellular cancer fraction (CCF) associated with that clone in that sample. We refer to these frequencies as the *inclusive clone frequencies*. Based on the clone definitions, we additionally define the *exclusive clone frequencies*,

$$x^\alpha = X^\alpha - \sum_{\beta \in \mathcal{D}(\alpha)} X^\beta \, . \tag{8}$$

Here $\mathcal{D}(\alpha)$ is the set of clones that are direct descendants of clone α, as defined by the tree $\mathcal{T}$. By this definition, x^α is a probability distribution, with $\sum_\alpha x^\alpha = 1$. We denote by **x** the ensemble of cluster size distributions for each of the phylogenies for a given tumor sample.

Genetic heterogeneity of tumor samples. We compute the heterogeneity of a tumor sample as the entropy of the clone frequency distribution,

$$S = \left\langle - \sum_{\alpha \in \mathcal{T}} x^\alpha \log x^\alpha \right\rangle_{\mathcal{T}} \, . \tag{9}$$

A higher entropy indicates a more diverse and less clonal tumor composition.

Distance between time points. The amount of evolution between the paired primary and recurrent tumor samples can be computed as the Kullback-Leibler divergence, which quantifies the amount of changes between the clones sizes between time points,

$$D_{\mathrm{KL}}(\mathbf{x}_{\mathrm{rec}} \| \mathbf{x}_{\mathrm{prim}}) = \left\langle \sum_{\alpha \in \mathcal{T}} x^\alpha_{\mathrm{rec}} \log \frac{x^\alpha_{\mathrm{rec}}}{x^\alpha_{\mathrm{prim}}} \right\rangle_{\mathcal{T}} \, . \tag{10}$$

To account for predictable evolution, i.e. concerning the fate of the clones present in the primary tumor, we disregard all clones α with inclusive clone frequency $X_\alpha < 0.03$; we observed that such clones are more likely to contain mutations with unobserved reads in the primary tumor and are introduced to the topology by the reconstruction algorithm by support of mutations in the recurrent tumors.

We define the clone frequencies of these shared clones shared between primary and recurrent tumors as:

$$\tilde{x}^\alpha = \begin{cases} x^\alpha / \tilde{Z}, & \text{if } X^\alpha_{\mathrm{prim}} \geq 0.03 \\ 0, & \text{otherwise,} \end{cases} \tag{11}$$

where $\tilde{Z} = \sum_{\alpha \in \mathcal{T} : X^\alpha_{\mathrm{prim}} \geq 0.03} x^\alpha$ is the normalization constant.

Fitness model for tumor clones

A fitness model is used to quantify the growth rates of clones. Here we propose a two-component model, which accounts for balancing selective pressures.

Immune fitness component. We quantify the negative selection on tumor clones imposed by the T cell recognition based on the neoantigen quality model as defined in eq. (1)

$$F_I^\alpha = \max_{(\mathbf{p}^{\mathrm{MT}},\, h) \in \mathcal{N}(\alpha)} Q(\mathbf{p}^{\mathrm{MT}}, h) \, , \tag{12}$$

where $\mathcal{N}(\alpha)$ is the set of neoantigens in clone α and their associated HLA alleles.

Driver gene component. We quantify selective advantage due to mutations in the recognized PDAC oncogenes, $\mathcal{O} = \{\mathrm{KRAS}, \mathrm{TP53}, \mathrm{CDKN2A}, \mathrm{SMAD4}\}$ by awarding each mutation from one of these genes,

$$F_P^\alpha = |\mathcal{G}(\alpha) \cap \mathcal{O}| \, , \tag{13}$$

where $\mathcal{G}(\alpha)$ is the set of genes mutated in clone α (including genes mutated in clones ancestral to α).

Combined fitness model. To account for negative selection due to immune recognition and positive selection of mutated oncogenes, we define an additive combined fitness model as

$$F^{\alpha}(\sigma_I, \sigma_P) = F_0 - \sigma_I F_I^{\alpha} + \sigma_P F_P^{\alpha}\ , \tag{14}$$

where $\sigma_I \geq 0$ and $\sigma_P \geq 0$ are weights assigning the amplitude to their respective fitness components; they also determine the total amplitude of selection described by the fitness model. Constant $F_0 \equiv F_0(\mathcal{T}, \sigma_I, \sigma_P)$ is a tree specific and clone independent constant determined by the normalization of clone frequencies for a given tumor sample and tree $\mathcal{T}$,

$$\sum_{\alpha \in \mathcal{T}} x^{\alpha} \exp\left(F^{\alpha}(\sigma_I, \sigma_P)\right) = 1, \tag{15}$$

giving $F_0 = -\log\left(\sum_{\alpha \in \mathcal{T}} x^{\alpha} \exp\left(-\sigma_I F_I^{\alpha} + \sigma_P F_P^{\alpha}\right)\right)$.

Tumor immune cost

For a given tumor, we compute its total immune fitness cost as the negative average of fitness, over clones in a given tree, and over the possible reconstructed trees,

$$\bar{F}_I = \left\langle \sum_{\alpha \in \mathcal{T}} x^{\alpha} F_I^{\alpha} \right\rangle_{\mathcal{T}}\ . \tag{16}$$

To evaluate the fitness of the new clones in the recurrent tumors, we compute an analogous average only over the clones that were not present in the primary tumor. As before, we use a 3% threshold on clone frequency,

$$\bar{F}_I^{\text{new}} = \left\langle \frac{\sum_{\alpha \in \mathcal{T}: X^{\alpha}_{\text{prim}} < 0.03} x^{\alpha}_{\text{rec}} F_I^{\alpha}}{\sum_{\alpha \in \mathcal{T}: X^{\alpha}_{\text{prim}} < 0.03} x^{\alpha}_{\text{rec}}} \right\rangle_{\mathcal{T}}\ . \tag{17}$$

Recurrent tumor clone composition predictions

For each primary and recurrent tumor pair, we predict the distribution of clone sizes in the recurrent tumor by fitness model projections from the primary tumor. In our model we combine the probability that a given clone in the primary tumor seeds a recurrence, together with a selective pressure as given by the fitness model. For a given clone α with a fitness F^{α}, the predicted exclusive clone frequency is

$$\hat{x}^{\alpha}_{\text{rec}}(\sigma_I, \sigma_P) = \tilde{x}^{\alpha}_{\text{prim}} \exp\left(F^{\alpha}(\sigma_I, \sigma_P)\right)\ , \tag{18}$$

and the inclusive frequency is

$$\hat{X}^{\alpha}_{\text{rec}}(\sigma_I, \sigma_P) = \sum_{\beta \in \mathcal{T}_{\alpha}} \tilde{x}^{\beta}_{\text{prim}} \exp\left(F^{\beta}(\sigma_I, \sigma_P)\right)\ , \tag{19}$$

where β iterates over all subclones of α ($\mathcal{T}_{\alpha}$ being a subtree of the tumor clone tree, rooted at clone α). We restrict predictions to clones that have been observed in the primary tumor, and we will use the shared clone frequency distributions as defined in eq. (11), both for the primary and recurrent tumors.

Neoantigen quality model fitting and model selection

The free parameters of the neoantigen quality model, $\Theta = \{a, k, w\}$, are trained on an independent cohort of 58 pancreatic cancer patients,[5] to optimize survival analysis log-rank score.[4,5] This cohort comprises samples from short and long term survivors and we have previously shown that the long-term survivors are likely to have increased immune activity in their tumors. We use our fitness model for tumor clones (14) to predict tumor growth in the pancreatic cancer patients. For each patient sample in the cohort we compute

$$\hat{n}(\sigma_I, \sigma_P, \Theta) = \sum_{\alpha} x^{\alpha}_{\text{prim}} \exp(F_{\alpha}(\sigma_I, \sigma_P)), \tag{20}$$

the predicted tumor population size. To limit the number of parameters, we fixed the slope parameter of the R component, $k = 1$.[5] The survival analysis is performed by splitting the patient cohort by the median value of

$n(\sigma_I, \sigma_P, \Theta)$ into high and low fitness groups and evaluation of the log-rank score, $S(\sigma_I, \sigma_P, \Theta)$ (multivariate log rank test from python package lifelines.statistics).

We computed the optimal parameters $\hat{\sigma}_I, \hat{\sigma}_P, \hat{\Theta}$ as an average $\langle(\sigma_I, \sigma_P, \Theta)\rangle_\rho$ over ρ, the probability distribution defined by the log-rank test score landscape for the cohort,

$$\rho(\sigma_I, \sigma_P, \Theta) = Z_\rho^{-1} \exp(S(\sigma_I, \sigma_P, \Theta)) \tag{21}$$

with Z_ρ the normalization constant assuring ρ is a probability distribution over the parameters with significant score, $\{(\sigma_I, \sigma_P, \Theta) : p(S(\sigma_I, \sigma_P, \Theta)) < 0.01\}$(p-values are computed with χ^2 test). This smoothing procedure is applied to select optimal parameters while preventing over-fitting on a potentially rugged score landscape. If no choice of parameters meets the significance threshold, we average the parameters that have the maximum observed value of the score.

The optimal value of parameter a, the midpoint of the logistic binding function R, is at $a = 22.9$ and the relative weight parameter for the two terms in component D eq. (7) is $w = 0.22$ (**Extended Data Table 1**). These are the parameter values we use to compute neoantigen qualities in the recurrent tumors cohort used in this study.

Model selection
Along with parameter training we performed a model selection effort to justify that all components of the fitness and neoantigen quality models are informative. We considered a variety of partial models and repeated the parameter training procedure via maximization of the the log-rank test score. We considered clone fitness model of single components only, namely the driver gene component- and the immune component-only,

$$F^\alpha(\sigma_P) = F_0 + \sigma_P F_P^\alpha \,, \tag{22}$$
$$F^\alpha(\sigma_I) = F_0 - \sigma_I F_I^\alpha \,. \tag{23}$$

Further we decomposed the immune fitness component by considering various variants of the neoantigen quality model:

$$Q(\mathbf{p}^{\mathsf{MT}}, h) = D(\mathbf{p}^{\mathsf{MT}}, \mathbf{p}^{\mathsf{WT}}, h) \,, \tag{24}$$
$$Q(\mathbf{p}^{\mathsf{MT}}, h) = R(\mathbf{p}^{\mathsf{MT}}) \,. \tag{25}$$

To compare the performance of the models of different complexities (number of fitted parameters), we computed the BIC[34] and AIC values (**Extended Data Table 1**). According to these criteria, the best performing model is our full clone fitness model with both the driver gene- and immune components, and the full neoantigen quality model.

Fitness model fitting and model selection
For a given pair of primary-recurrent tumor samples from a given patient, we fit the fitness model parameters, σ_I, σ_P, to minimize the Kullback-Leibler divergence between the predicted clone composition and the observed clone composition of the recurrent tumor sample,

$$D_{\mathsf{KL}}(\tilde{\mathbf{x}}_{\mathsf{rec}} \| \hat{\mathbf{x}}_{\mathsf{rec}}) = \min_{\sigma_I, \sigma_P \geq 0} \left\langle \sum_{\alpha \in \mathcal{T}} \tilde{x}_{\mathsf{rec}}^\alpha \log \frac{\tilde{x}_{\mathsf{rec}}^\alpha}{\hat{x}_{\mathsf{rec}}^\alpha(\sigma_I, \sigma_P)} \right\rangle_{\mathcal{T}} . \tag{26}$$

The likelihood that the observed distributions are samples of populations with the predicted clone frequencies takes the form (by Sanov's theorem[51])

$$L \sim \exp\left(-n\, D_{\mathsf{KL}}(\tilde{\mathbf{x}}_{\mathsf{rec}} \| \hat{\mathbf{x}}_{\mathsf{rec}})\right), \tag{27}$$

where n is a factor standing for the effective population size of the cells in the recurrent tumor sample, from which the clone frequencies were inferred. The effective population size reflects the sampling error and our ability to correctly estimate clone frequencies from the bulk sequencing data. It depends on multiple factors, such as the sequencing depth, the purity of the sample, and the phylogeny reconstruction algorithm. We estimate the effective population size for each sample, as described in a following section.

We evaluate the likelihood L_0 under the null model of neutral evolution, which assigns fitness values $F_N^\alpha = 0$ to all clones and predicts clade frequencies $\hat{\mathbf{x}}_{\text{rec}} = \tilde{\mathbf{x}}_{\text{prim}}$. The likelihood of the data under this model is, analogously to (27), given by

$$L_0 \sim \exp\left(-n\, D_{\text{KL}}(\tilde{\mathbf{x}}_{\text{prim}} \| \tilde{\mathbf{x}}_{\text{rec}})\right) \ . \tag{28}$$

To compare models of varying complexity, we compute the Bayesian Information Criterion (BIC),[34]

$$\text{BIC}(L) = |\Theta| \log(n) - 2\log(L) \ , \tag{29}$$

where Θ is the set of optimized parameters, $|\Theta| = 2$, arriving at an adjusted log-likelihood,

$$\log(L^{\text{adj}}) \sim -\text{BIC}(L)/2 = \log(L) - \log(n) \ . \tag{30}$$

To assess the predictive power of individual fitness model components, we consider partial models and their corresponding optimized likelihoods: the immune component only model $F_I^\alpha(\sigma_I) \equiv F^\alpha(\sigma_I, \sigma_P = 0)$, with likelihood L_I; and the driver gene component only model $F_P^\alpha(\sigma_P) \equiv F^\alpha(\sigma_I = 0, \sigma_P)$ with likelihood L_P. Each of these models has one free parameter, we apply the BIC-based correction (30) to compute the adjusted log-likelihoods $\log(L_I^{\text{adj}}) = \log(L_I) - \log(n)/2$ and $\log(L_P^{\text{adj}}) = \log(L_P) - \log(n)/2$.

In general, to compare fitting of alternative models F_1 and F_2 on a cohort S, for each sample s in the cohort we compute the log-likelihood score, $\Delta\ell(s, F_1, F_2) = \log(L_1^{\text{adj}}(s)) - \log(L_2^{\text{adj}}(s))$. We also evaluate the aggregated score over samples in cohort $\mathcal{S}$,

$$\Delta\mathcal{L}^{\mathcal{S}}(F_1, F_2) = \sum_{s \in \mathcal{S}} \Delta\ell(s, F_1, F_2) \ , \tag{31}$$

where s iterates over samples in the cohort. Positive scores favor model L_1 over model L_2.

Effective cancer cell population size n
To account for sampling error which affects clone frequency inference, we estimate the error of mutation frequencies for each of the tumor samples in our data. We evaluate frequencies for each mutation m in a given sample s, with the frequencies from the individual trees $\mathcal{T}$, given by $x(m) = X^\alpha$ where m originates in clone $\alpha \in \mathcal{T}$. The variance is computed over the 5 trees reconstructed for that sample, $\sigma^2(x_m) = \langle x(m)^2 \rangle_{\mathcal{T}} - \langle x(m) \rangle_{\mathcal{T}}^2$. The effective size n for a given sample scales proportionally with the inverse of variance, giving our estimate of n as

$$n(s) \sim \frac{1}{\langle \sigma^2(x_m) \rangle_{m \in s}} \ . \tag{32}$$

For the patients with multiple samples the variance of mutation frequencies from tree reconstruction is reduced due to information from other samples; to account for this effect we divide $n(s)$ by the total number of additional samples. The estimated effective cancer population sizes vary from 79.79 to 1189.60, with a mean of 187.23 and median 244.6.

Clone fitness model selection
We compute the log-likelihood score for the alternative models for each recurrent tumor sample (**Extended Data Table 1**). In particular, we observe that 19 out of 22 LTS samples (86%) and 17 our of 33 (52%) are better described by the model with selection, F, rather than the null model, $F_N \equiv 0$ (giving $\Delta\ell(s, F, F_N) > 0$). Evaluating the aggregated log-likelihood score on the LTS and STS cohorts, we observe evidence for the model with selection in both cohorts, $\Delta\mathcal{L}^{\text{LTS}}(F, F_N) = 1241$ nats and $\Delta\mathcal{L}^{\text{STS}}(F, F_N) = 198$ nats , with a mean of 56.42 nats and 6.01 nats respectively. The fit, and therefore the predictive power of model F, is relatively stronger in the LTS cohort.

We assess that the oncogenic selection, described by the driver gene component model F_P, provides predictive signal on its own, with 14 out of 22 LTS samples (64%) and 13 out of 33 STS samples (39%) having positive log-likelihood score, $\Delta\ell(s, F_P, F_N) > 0$. Evaluating the aggregated log-likelihood score on the LTS and STS cohorts, we observe evidence for the model with oncogenic selection in both cohorts, $\Delta\mathcal{L}^{\text{LTS}}(F_P, F_N) = 1041$ nats and $\Delta\mathcal{L}^{\text{STS}}(F_P, F_N) = 223$ nats, with a mean of 47.34 nats and 6.77 nats respectively. Again, the fit of the partial model is stronger in the LTS cohort. This effect could be explained indirectly by the negative immune selection, which reduces tumor heterogeneity and facilitates clonal composition predictions in the LTS cohort.

The immune component F_I on its own has less predictive power, with positive log-likelihood score $\Delta\ell(s, F_I, F_N)$ for 11 out of 22 LTS samples (50%) and only 4 out of 33 STS samples (12%). The aggregated log-likelihood score values are $\Delta\mathcal{L}^{\text{LTS}}(F_I, F_N) = 579$ nats (mean of 26.31 nats) and $\Delta\mathcal{L}^{\text{STS}}(F_I, F_N) = 91$ nats (mean of 2.75 nats).

The full model with both components provides an improvement to the driver-gene component-only model for 11 out of 22 LTS samples (50%) but only 5 out of 33 STS samples (15%), as quantified by $\Delta\ell(s, F, F_P) > 0$. The aggregated log-likelihood score on the LTS and STS cohorts are $\Delta\mathcal{L}^{\text{LTS}}(F, F_P) = 200$ nats and $\Delta\mathcal{L}^{\text{STS}}(F, F_P) = -25$ nats respectively. This results means that inclusion of the immune component directly improves prediction of clone dynamics in the LTS cohort, but it does not for the STS cohort. All these results are reported in **Extended Data Table 1b** and in **Extended Data Figure 9**.

Accuracy of clone growth fitting
We consider the observed and model fitted clone frequency changes, $X^{\alpha}_{\text{rec}}/X^{\alpha}_{\text{prim}}$ and $\hat{X}^{\alpha}_{\text{rec}}/X^{\alpha}_{\text{prim}}$ across all clones in all tumors in the cohorts. For a given cohort we define the accuracy of a model as the fraction of clones for which the direction of change is the same, i.e. ($X^{\alpha}_{\text{rec}}/X^{\alpha}_{\text{prim}} > 1$ and $\hat{X}^{\alpha}_{\text{rec}}/X^{\alpha}_{\text{prim}} > 1$) or ($X^{\alpha}_{\text{rec}}/X^{\alpha}_{\text{prim}} < 1$ and $\hat{X}^{\alpha}_{\text{rec}}/X^{\alpha}_{\text{prim}} < 1$) or ($X^{\alpha}_{\text{rec}}/X^{\alpha}_{\text{prim}} = 1$ and $\hat{X}^{\alpha}_{\text{rec}}/X^{\alpha}_{\text{prim}} = 1$). We consider all clones with frequency larger than 0.03 in the primary tumor, from the top scoring trees for each patient. We obtain accuracy of 71% over 243 clones in the LTS cohort, and 58% over 389 clones in the STS cohort. The Pearson correlation coefficients are $r^{\text{LTS}} = 0.57$ and $r^{\text{STS}} = 0.35$ (as computed on log-transformed frequency changes, $\log X^{\alpha}_{\text{rec}}/X^{\alpha}_{\text{prim}}$ and $\log \hat{X}^{\alpha}_{\text{rec}}/X^{\alpha}_{\text{prim}}$) and Spearman rank coefficients are $\rho^{\text{LTS}} = 0.65$ and $\rho^{\text{STS}} = 0.28$ (**Extended Data Table 1b**).

TCR beta (TCRB) sequencing
We extracted genomic DNA from n = 23 primary and recurrent STS and LTS PDACs according to the manufacturer's instructions (QIAsymphony, Qiagen). We verified the quantity and quality of extracted DNA before sequencing. We then used a standard quantity of input DNA, amplified and sequenced the CDR3β regions using the survey multiplexed PCR ImmunoSeq assay (Adaptive Biotechnologies). The ImmunoSeq platform combines multiplex PCR with high-throughput sequencing to selectively amplify the rearranged complementarity-determining region 3 (CDR3β) of the TCR, producing fragments sufficiently long to identify the VDJ region spanning each unique CDR3β. After correcting for sequencing coverage, PCR bias, primer bias, and sequencing errors, we define a T cell clone as a T cell with a unique TCRB CDR3β amino acid sequence.

Dissimilarity index to estimate antigen-specificity in a T cell repertoire
To estimate the antigen-specificity of a T cell repertoire, for each repertoire, we apply a sequence based probabilistic model called a Restricted Boltzmann Machine (RBM).[18] The RBM model assigns a probabilistic score of an antigen specific response to each T cell clone in a sample, based on the frequency and the CDR3β sequence similarity of the top 25 ranking clones. Based on these RBM scores for each clone, we estimate for each repertoire, a TCR dissimilarity index $DI = \frac{1}{f}$ where:

$$f = \frac{1}{T} \sum_{i<j} e^{-\left(\frac{d(\sigma_i, \sigma_j)}{\delta}\right)^2} \tag{33}$$

where T is the total number of terms in the sum ($T = M(M-1)/2$), $M = 25$ (the top 25 clones in the repertoire with the highest RBM scores), and $d(\sigma_i, \sigma_j)$ is a distance obtained from the global pairwise alignment score between the CDR3β amino acid sequences σ_i and σ_j. This score is computed using the BLOSUM62 matrix corrected with an offset such that all its weights are positive, $-S(A, B) + \max_{A,B}(S(A, B)) \geq 0$, where $S(A, B)$ are the usual BLOSUM62 matrix elements. The parameter δ represents a typical scale of the BLOSUM-weighted distance d and it is set to δ=9.37, the average distance d between reported epitope-specific CDR3β sequences (we use an influenza-specific repertoire[21]). As a control, we calculate this TCR dissimilarity index between the top 25 clones in the repertoire based on clone size (TCR dissimilarity index $-$ clone size), and not the RBM computed probability (**Extended Data Figure 1c**).

To verify that the difference in the TCR dissimilarity index between LTSs and STSs is robust, we randomly subsample the repertoire down to a few hundred clones and repeat the RBM training, score assignment, and TCR dissimilarity index estimation 10 times (**Extended Data Figure 1b**).

Statistics

Survival curves were compared using log-rank test (Mantel-Cox). Comparison between two groups was performed using unpaired two-tailed Mann-Whitney test, or Wald's test for gene expression analyses to correct for multiple comparison testing. Correlation between two variables was performed using two-tailed Pearson correlation. Categorical variables were compared using chi-square test. Probability distributions were compared using two-sided Kolmogorov-Smirnov (KS) test. All comparison groups had equivalent variances. $P < 0.05$ was considered to be statistically significant. Data analysis was performed using statistical software (Prism 7.0, GraphPad Software v.9.1.0 and Python v.3.4).

References

[1] Shankaran, V. *et al.* Pillars article: Ifngamma and lymphocytes prevent primary tumour development and shape tumour immunogenicity. nature. 2001. 410: 1107-1111. *J Immunol* **201**, 827–831 (2018). URL `https://www.ncbi.nlm.nih.gov/pubmed/30038035`.

[2] Hanahan, D. & Weinberg, R. A. Hallmarks of cancer: the next generation. *Cell* **144**, 646–74 (2011). URL `https://www.ncbi.nlm.nih.gov/pubmed/21376230`.

[3] Matsushita, H. *et al.* Cancer exome analysis reveals a t-cell-dependent mechanism of cancer immunoediting. *Nature* **482**, 400–4 (2012). URL `https://www.ncbi.nlm.nih.gov/pubmed/22318521`.

[4] Łuksza, M. *et al.* A neoantigen fitness model predicts tumour response to checkpoint blockade immunotherapy. *Nature* **551**, 517–520 (2017).

[5] Balachandran, V. P. *et al.* Identification of unique neoantigen qualities in long-term survivors of pancreatic cancer. *Nature* **551**, 512–516 (2017).

[6] Burnet, F. M. The concept of immunological surveillance. *Prog Exp Tumor Res* **13**, 1–27 (1970). URL `https://www.ncbi.nlm.nih.gov/pubmed/4921480`.

[7] Dunn, G. P., Bruce, A. T., Ikeda, H., Old, L. J. & Schreiber, R. D. Cancer immunoediting: from immunosurveillance to tumor escape. *Nat Immunol* **3**, 991–8 (2002). URL `https://www.ncbi.nlm.nih.gov/pubmed/12407406`.

[8] Schumacher, T. N. & Schreiber, R. D. Neoantigens in cancer immunotherapy. *Science* **348**, 69–74 (2015). URL `https://www.ncbi.nlm.nih.gov/pubmed/25838375`.

[9] Rosenthal, R. *et al.* Neoantigen-directed immune escape in lung cancer evolution. *Nature* **567**, 479–485 (2019). URL `https://www.ncbi.nlm.nih.gov/pubmed/30894752`.

[10] Zhang, A. W. *et al.* Interfaces of malignant and immunologic clonal dynamics in ovarian cancer. *Cell* **173**, 1755–1769 e22 (2018). URL `https://www.ncbi.nlm.nih.gov/pubmed/29754820`.

[11] Jimenez-Sanchez, A. *et al.* Heterogeneous tumor-immune microenvironments among differentially growing metastases in an ovarian cancer patient. *Cell* **170**, 927–938 e20 (2017). URL `https://www.ncbi.nlm.nih.gov/pubmed/28841418`.

[12] Balli, D., Rech, A. J., Stanger, B. Z. & Vonderheide, R. H. Immune cytolytic activity stratifies molecular subsets of human pancreatic cancer. *Clin Cancer Res* **23**, 3129–3138 (2017). URL `https://www.ncbi.nlm.nih.gov/pubmed/28007776`.

[13] Rizvi, N. A. *et al.* Cancer immunology. mutational landscape determines sensitivity to pd-1 blockade in non-small cell lung cancer. *Science* **348**, 124–8 (2015). URL `https://www.ncbi.nlm.nih.gov/pubmed/25765070`.

[14] Van Allen, E. M. *et al.* Genomic correlates of response to ctla-4 blockade in metastatic melanoma. *Science* **350**, 207–211 (2015). URL `https://www.ncbi.nlm.nih.gov/pubmed/26359337`.

[15] Yachida, S. *et al.* Distant metastasis occurs late during the genetic evolution of pancreatic cancer. *Nature* **467**, 1114–7 (2010). URL `https://www.ncbi.nlm.nih.gov/pubmed/20981102`.

[16] Ino, Y. *et al.* Immune cell infiltration as an indicator of the immune microenvironment of pancreatic cancer. *British Journal of Cancer* **108**, 914–923 (2013). URL `https://doi.org/10.1038/bjc.2013.32`.

[17] Riquelme, E. *et al.* Tumor microbiome diversity and composition influence pancreatic cancer outcomes. *Cell* **178**, 795–806 e12 (2019). URL `https://www.ncbi.nlm.nih.gov/pubmed/31398337`.

[18] Bravi, B. *et al.* Probing t-cell response by sequence-based probabilistic modeling. *PLoS Comput Biol* **17**, e1009297 (2021). URL `https://www.ncbi.nlm.nih.gov/pubmed/34473697`.

[19] Sakamoto, H. *et al.* The evolutionary origins of recurrent pancreatic cancer. *Cancer Discov* **10**, 792–805 (2020). URL `https://www.ncbi.nlm.nih.gov/pubmed/32193223`.

[20] Dyall, R. *et al.* Heteroclitic immunization induces tumor immunity. *J Exp Med* **188**, 1553–61 (1998). URL `https://www.ncbi.nlm.nih.gov/pubmed/9802967`.

[21] Dash, P. *et al.* Quantifiable predictive features define epitope-specific t cell receptor repertoires. *Nature* **547**, 89–93 (2017). URL `https://www.ncbi.nlm.nih.gov/pubmed/28636592`.

[22] Glanville, J. *et al.* Identifying specificity groups in the t cell receptor repertoire. *Nature* **547**, 94–98 (2017). URL `https://www.ncbi.nlm.nih.gov/pubmed/28636589`.

[23] Birnbaum, M. E. *et al.* Deconstructing the peptide-mhc specificity of t cell recognition. *Cell* **157**, 1073–87 (2014). URL `https://www.ncbi.nlm.nih.gov/pubmed/24855945`.

[24] Solache, A. *et al.* Identification of three hla-a*0201-restricted cytotoxic t cell epitopes in the cytomegalovirus protein pp65 that are conserved between eight strains of the virus. *J Immunol* **163**, 5512–8 (1999). URL `https://www.ncbi.nlm.nih.gov/pubmed/10553078`.

[25] Kawakami, Y. *et al.* Recognition of multiple epitopes in the human melanoma antigen gp100 by tumor-infiltrating t lymphocytes associated with in vivo tumor regression. *J Immunol* **154**, 3961–8 (1995). URL `https://www.ncbi.nlm.nih.gov/pubmed/7706734`.

[26] Parkhurst, M. R. *et al.* Improved induction of melanoma-reactive ctl with peptides from the melanoma antigen gp100 modified at hla-a*0201-binding residues. *J Immunol* **157**, 2539–48 (1996). URL `https://www.ncbi.nlm.nih.gov/pubmed/8805655`.

[27] Capietto, A. H. *et al.* Mutation position is an important determinant for predicting cancer neoantigens. *J Exp Med* **217** (2020). URL `https://www.ncbi.nlm.nih.gov/pubmed/31940002`.

[28] Deshwar, A. G. *et al.* Phylowgs: reconstructing subclonal composition and evolution from whole-genome sequencing of tumors. *Genome Biol* **16**, 35 (2015). URL `https://www.ncbi.nlm.nih.gov/pubmed/25786235`.

[29] Evans, R. A. *et al.* Lack of immunoediting in murine pancreatic cancer reversed with neoantigen. *JCI Insight* **1** (2016). URL `https://www.ncbi.nlm.nih.gov/pubmed/27642636`.

[30] Freed-Pastor, W. A. *et al.* Abstract pr14: Preclinical models to dissect immune escape in pancreatic cancer. *Cancer Research* **79**, PR14–PR14 (2019).

[31] Barthel, F. P. *et al.* Longitudinal molecular trajectories of diffuse glioma in adults. *Nature* **576**, 112–120 (2019). URL `https://www.ncbi.nlm.nih.gov/pubmed/31748746`.

[32] Zaretsky, J. M. *et al.* Mutations associated with acquired resistance to pd-1 blockade in melanoma. *N Engl J Med* **375**, 819–29 (2016). URL `https://www.ncbi.nlm.nih.gov/pubmed/27433843`.

[33] Cancer Genome Atlas Research Network. Electronic address, a. a. d. h. e. & Cancer Genome Atlas Research, N. Integrated genomic characterization of pancreatic ductal adenocarcinoma. *Cancer Cell* **32**, 185–203 e13 (2017). URL `https://www.ncbi.nlm.nih.gov/pubmed/28810144`.

[34] Schwarz, G. Estimating the dimension of a model. *The Annals of Statistics* **6**, 461–464 (1978). URL `http://www.jstor.org/stable/2958889`.

[35] Cibulskis, K. *et al.* Sensitive detection of somatic point mutations in impure and heterogeneous cancer samples. *Nat Biotechnol* **31**, 213–9 (2013). URL `https://www.ncbi.nlm.nih.gov/pubmed/23396013`.

[36] Saunders, C. T. *et al.* Strelka: accurate somatic small-variant calling from sequenced tumor-normal sample pairs. *Bioinformatics* **28**, 1811–7 (2012). URL `https://www.ncbi.nlm.nih.gov/pubmed/22581179`.

[37] Tate, J. G. *et al.* Cosmic: the catalogue of somatic mutations in cancer. *Nucleic Acids Res* **47**, D941–D947 (2019). URL `https://www.ncbi.nlm.nih.gov/pubmed/30371878`.

[38] Robinson, J. T., Thorvaldsdottir, H., Wenger, A. M., Zehir, A. & Mesirov, J. P. Variant review with the integrative genomics viewer. *Cancer Res* **77**, e31–e34 (2017). URL https://www.ncbi.nlm.nih.gov/pubmed/29092934.

[39] Grossman, R. L. *et al.* Toward a shared vision for cancer genomic data. *N Engl J Med* **375**, 1109–12 (2016). URL https://www.ncbi.nlm.nih.gov/pubmed/27653561.

[40] Szolek, A. *et al.* Optitype: precision hla typing from next-generation sequencing data. *Bioinformatics* **30**, 3310–6 (2014). URL https://www.ncbi.nlm.nih.gov/pubmed/25143287.

[41] Cingolani, P. *et al.* A program for annotating and predicting the effects of single nucleotide polymorphisms, snpeff: Snps in the genome of drosophila melanogaster strain w1118; iso-2; iso-3. *Fly* **6**, 80–92 (2012).

[42] Nielsen, M. *et al.* Reliable prediction of t-cell epitopes using neural networks with novel sequence representations. *Protein Sci* **12**, 1007–17 (2003). URL https://www.ncbi.nlm.nih.gov/pubmed/12717023.

[43] Lundegaard, C. *et al.* Netmhc-3.0: accurate web accessible predictions of human, mouse and monkey mhc class i affinities for peptides of length 8-11. *Nucleic Acids Res* **36**, W509–12 (2008). URL https://www.ncbi.nlm.nih.gov/pubmed/18463140.

[44] Gallardo, H. F., Tan, C., Ory, D. & Sadelain, M. Recombinant retroviruses pseudotyped with the vesicular stomatitis virus g glycoprotein mediate both stable gene transfer and pseudotransduction in human peripheral blood lymphocytes. *Blood* **90**, 952–7 (1997). URL https://www.ncbi.nlm.nih.gov/pubmed/9242523.

[45] Ghani, K. *et al.* Efficient human hematopoietic cell transduction using rd114- and galv-pseudotyped retroviral vectors produced in suspension and serum-free media. *Hum Gene Ther* **20**, 966–74 (2009). URL https://www.ncbi.nlm.nih.gov/pubmed/19453219.

[46] Gros, A. *et al.* Prospective identification of neoantigen-specific lymphocytes in the peripheral blood of melanoma patients. *Nat Med* **22**, 433–8 (2016). URL https://www.ncbi.nlm.nih.gov/pubmed/26901407.

[47] Cohen, C. J., Zhao, Y., Zheng, Z., Rosenberg, S. A. & Morgan, R. A. Enhanced antitumor activity of murine-human hybrid t-cell receptor (tcr) in human lymphocytes is associated with improved pairing and tcr/cd3 stability. *Cancer Res* **66**, 8878–86 (2006). URL https://www.ncbi.nlm.nih.gov/pubmed/16951205.

[48] Riviere, I., Brose, K. & Mulligan, R. C. Effects of retroviral vector design on expression of human adenosine deaminase in murine bone marrow transplant recipients engrafted with genetically modified cells. *Proc Natl Acad Sci U S A* **92**, 6733–7 (1995). URL https://www.ncbi.nlm.nih.gov/pubmed/7624312.

[49] Vita, R. *et al.* The immune epitope database (iedb) 3.0. *Nucleic acids research* **43**, D405–D412 (2014).

[50] Andreatta, M. & Nielsen, M. Gapped sequence alignment using artificial neural networks: application to the mhc class i system. *Bioinformatics* **32**, 511–7 (2016). URL https://www.ncbi.nlm.nih.gov/pubmed/26515819.

[51] Cover, T. M. & Thomas, J. A. *Elements of Information Theory (Wiley Series in Telecommunications and Signal Processing)* (Wiley-Interscience, USA, 2006).
