# Supplementary material for: Neoantigen quality predicts immunoediting in survivors of pancreatic cancer
Source: Nature. 2022 May 19;606(7913):389–95. doi: 10.1038/s41586-022-04735-9 (PMC9177421; doi:10.1038/s41586-022-04735-9)
Supplement: Supplementary file 2 — Reporting Summary [file 41586_2022_4735_MOESM2_ESM.pdf]

nature research

Corresponding author(s): Marta Luksza, Benjamin Greenbaum, Vinod Balachandran

Last updated by author(s): Mar 22, 2022

Reporting Summary

Nature Research wishes to improve the reproducibility of the work that we publish. This form provides structure for consistency and transparency in reporting. For further information on Nature Research policies, see our Editorial Policies and the Editorial Policy Checklist.

Statistics

For all statistical analyses, confirm that the following items are present in the figure legend, table legend, main text, or Methods section.

n/a	Confirmed	
☐	☒	The exact sample size (*n*) for each experimental group/condition, given as a discrete number and unit of measurement
☐	☒	A statement on whether measurements were taken from distinct samples or whether the same sample was measured repeatedly
☐	☒	The statistical test(s) used AND whether they are one- or two-sided<br>*Only common tests should be described solely by name; describe more complex techniques in the Methods section.*
☐	☒	A description of all covariates tested
☐	☒	A description of any assumptions or corrections, such as tests of normality and adjustment for multiple comparisons
☐	☒	A full description of the statistical parameters including central tendency (e.g. means) or other basic estimates (e.g. regression coefficient) AND variation (e.g. standard deviation) or associated estimates of uncertainty (e.g. confidence intervals)
☐	☒	For null hypothesis testing, the test statistic (e.g. *F*, *t*, *r*) with confidence intervals, effect sizes, degrees of freedom and *P* value noted<br>*Give P values as exact values whenever suitable.*
☐	☒	For Bayesian analysis, information on the choice of priors and Markov chain Monte Carlo settings
☒	☐	For hierarchical and complex designs, identification of the appropriate level for tests and full reporting of outcomes
☐	☒	Estimates of effect sizes (e.g. Cohen's *d*, Pearson's *r*), indicating how they were calculated

Our web collection on statistics for biologists contains articles on many of the points above.

Software and code

Policy information about availability of computer code

Data collection

Flow cytometric data were collected using FACSDiva (BD Biosciences, version 8.0.1).

Code used to construct and apply the model is available at https://github.com/LukszaLab/NeoantigenEditing.

Data analysis

All data analysis was performed using Prism 7.0, GraphPad Software v.9.1.0, or Python v3.4 unless otherwise indicated.

For WES, sequence data were demultiplexed using Illumina CASAVAsoftware. Reads were aligned to the reference human genome (hg19) using the Burrows-Wheeler Alignment tool (bwa mem v0.7.17) and samtools (v1.6). Duplicates were marked with picard-2.11.0 MarkDuplicates (http://broadinstitute.github.io/picard). Indel realignments were done with the Genome Analysis toolkit (GenomeAnalysisTK-3.8-1-0-gf15c1c3ef) RealignerTargetCreator and IndelRealigner (ref #35) using 1000 genome phase1 indel (1000G_phase1.indels.b37.vcf) and Mills indel calls (Mills_and_1000G_gold_standard.indels.b37.vcf) as references. Base calls were recalibrated with BaseRecalibrator (ref #35) and dbSNP version 138.

Mutations were called using Mutect 1.1.7 (ref #35) (https://software.broadinstitute.org/gatk/download/mutect) and Strelka 1.0.15 (ref #36). Unbiased normal/tumor read counts for each SNV and indel call were assigned with the bam-readcount software 0.8.0-unstable-6-963acab-dirty (commit 963acab-dirty) (https://github.com/genome/bam-readcount).

HLA-I typing for PDAC patients was performed in silico with the OptiType version 1.3.3 tool (https://github.com/FRED-2/OptiType) (ref #40).

Tumor clones were reconstructed with the PhyloWGS algorithm (https://github.com/morrislab/phylowgs) (ref #28).

For neoantigen prediction, wild-type and mutant genomic sequences corresponding to coding mutations were translated to an amino acid sequence consistent with the GRCh37 reference genome (GRCh37.75) using snpEff.v4.3t software (ref #41). Predictions of MHC class-I binding for both the WT peptide (pWT) and mutant peptide (pMT) were estimated using the NetMHC 3.4 software (ref #42, 43)

We inferred R using a set of known positive epitopes derived from the IEDB (ref #49), restricting the search to all human infectious disease class-I restricted targets with positive immune assays (http://www.iedb.org). To calculate the alignments between all neoantigens and IEDB epitope sequences, we used the BLOSUM62 matrix (gap opening penalty=-11, gap extention penalty=-1) with the blastp algorithm. We calculated alignment scores with the Biopython Bio.pairwise2 package (http://biopython.org) for all alignments identified with blastp.

To estimate the antigen-specificity of a T cell repertoire, for each repertoire, we apply a sequence based probabilistic model called a Restricted Boltzmann Machine (RBM) (ref #18). The RBM model is trained on the sampled CDR3b sequences and their abundance. Once the parameters are fixed, it allows us to assign probabilistic scores of specific response to each T cell clone in the sample. We considered the top 25 ranking clones according to this score, and estimated a CDR3b sequence dissimilarity index (DI) within this set.

For manuscripts utilizing custom algorithms or software that are central to the research but not yet described in published literature, software must be made available to editors and reviewers. We strongly encourage code deposition in a community repository (e.g. GitHub). See the Nature Research guidelines for submitting code & software for further information.

Data

Policy information about availability of data

All manuscripts must include a data availability statement. This statement should provide the following information, where applicable:

- Accession codes, unique identifiers, or web links for publicly available datasets
- A list of figures that have associated raw data
- A description of any restrictions on data availability

Source data are provided for all experiments. All raw sequencing data obtained through the Johns Hopkins Hospital medical donation program have been previously described (ref #19) and are available in the European Genome–Phenome Archive under accession number EGAS00001004097. All other raw sequencing data are available in the NCBI Sequence Read Archive under accession number PRJNA648923 (https://dataview.ncbi.nlm.nih.gov/object/PRJNA648923?reviewer=5hj966ftr4pnjbslhremst8mln). The ICGC data used in this study are available at https://dcc.icgc.org/repositories under the identifier PACA-AU. The TCGA data used in this study are from TCGA-PAAD dataset available at the NCI Genomic Data Commons (https://gdc.cancer.gov/).

Field-specific reporting

Please select the one below that is the best fit for your research. If you are not sure, read the appropriate sections before making your selection.

☒ Life sciences ☐ Behavioural & social sciences ☐ Ecological, evolutionary & environmental sciences

For a reference copy of the document with all sections, see nature.com/documents/nr-reporting-summary-flat.pdf

Life sciences study design

All studies must disclose on these points even when the disclosure is negative.

Sample size	Sample sizes were determined based on a priori estimated sizes required to detected differences in evolutionary patterns. We were unable to calculate pre-specified effect sizes to optimally determine sample sizes in the two groups, given no prior reports examine the long-term tumor evolution in humans with pancreatic ductal adenocarcinoma.
Data exclusions	Samples were either primary and recurrent pancreatic ductal adenocarcinoma tissues obtained through rapid autopsy, or surgical resection. We excluded adenocarcinomas in cystic pancreatic neoplasms and neuroendocrine tumors given their different genetic, histological, and clinical features compared to PDAC, the most common type of pancreatic cancer. All data exclusions were pre-established at the outset of the study.
Replication	TCR cross reactivity experiments were replicated across 3 different epitope strengths and were reproducible. All other experiments were observational in patient samples with the indicated sample sizes.
Randomization	There was no randomization. Covariates were controlled by matching primary tumors in short and long-term survivors by similar clinicopathological characteristics (Supplementary Table 1).
Blinding	Investigators were blinded to the allocated groups during data collection (whole exome sequencing, computational mutation and neoantigen prediction). After data were collected, the analysis was performed unblinded to accurately interpret the results.

Reporting for specific materials, systems and methods

We require information from authors about some types of materials, experimental systems and methods used in many studies. Here, indicate whether each material, system or method listed is relevant to your study. If you are not sure if a list item applies to your research, read the appropriate section before selecting a response.

Materials & experimental systems

n/a	Involved in the study	
☐	☒	Antibodies
☐	☒	Eukaryotic cell lines
☒	☐	Palaeontology and archaeology
☒	☐	Animals and other organisms
☐	☒	Human research participants
☒	☐	Clinical data
☒	☐	Dual use research of concern

Methods

n/a	Involved in the study	
☒	☐	ChIP-seq
☐	☒	Flow cytometry
☒	☐	MRI-based neuroimaging

Antibodies

Antibodies used

Flow cytometry:
CD3 - clone SK-7, PE-Cy7 (Biolegend Cat# 344816); 4 µl/sample
CD8 - clone SK1, Alexa Fluor 700 (Biolegend Cat# 344724); 2 µl/sample
mTRB - clone H57-597, PE-Cy5 (Biolegend Cat# 109210); 0.5 µl/sample
CD137 - clone 4B4-1, PE (Biolegend Cat# 309804); 3 µl/sample

Validation

All antibodies were validated by the manufacturer and used per their instructions. In our experiments, isotype and/or FMO control samples were included. Additional information on validation can be found on the manufacturers' websites:

CD3 - clone SK-7, PE-Cy7 (Biolegend Cat# 344816): https://www.biolegend.com/en-gb/products/pe-cyanine7-anti-human-cd3-antibody-6934?GroupID=BLG5900
CD8 - clone SK1, Alexa Fluor 700 (Biolegend Cat# 344724): https://www.biolegend.com/en-us/search-results/alexa-fluor-700-anti-human-cd8-antibody-9062?GroupID=BLG10167
mTRB - clone H57-597, PE-Cy5 (Biolegend Cat# 109210): https://www.biolegend.com/en-us/search-results/pe-cyanine5-anti-mouse-tcr-beta-chain-antibody-273
CD137 - clone 4B4-1, PE (Biolegend Cat# 309804): https://www.biolegend.com/en-us/search-results/pe-anti-human-cd137-4-1bb-antibody-1510

Eukaryotic cell lines

Policy information about cell lines

Cell line source(s)

T2 were a kind gift from Michel Sadelain. H29 cells were developed in the Richard C. Mulligan lab and have been previously described (PMID: 8876147). The K562 cell line was purchased from ATCC (CCL-243).

Authentication

We authenticated T2 cells based on concentration-dependent surface upregulation of HLA-A2 with exogenous HLA-A2-restricted peptides. H29 cells were strictly maintained with two selection antibiotics: G418 (gag/pol selection) and puromycin (VSV-G selection) to ensure maintenance of retroviral protein-expressing plasmids. STR profiling was performed to authenticate the K562 cell line.

Mycoplasma contamination

Cell lines were regularly tested using MycoAlert Mycoplasma Detection Kit (Lonza). None of the cell lines used in this study tested positive for Mycoplasma.

Commonly misidentified lines
(See ICLAC register)

No commonly misidentified lines were used in this study.

Human research participants

Policy information about studies involving human research participants

Population characteristics

We collected matched primary and recurrent PDACs through surgical resection at Memorial Sloan Kettering Cancer Center (MSK) (n = 5/9 LTS), and the Garvan Institute of Medical Research (n = 1/9 LTS) (Supplementary Table 1). Additional matched primary and recurrent PDACs were previously obtained through the Gastrointestinal Cancer Rapid Medical Donation Program at The Johns Hopkins Hospital (JHH) (n = 3/9 LTS, 6/6 STS) and have been described (ref # 19) (Supplementary Table 1). Cohorts of primary only PDAC were previously collected at MSK (MSK primary PDAC cohort) and the International Cancer Genome Consortium (ICGC primary PDAC cohort) through surgical resection as described (ref # 5, 33).

Recruitment

All patients with pancreatic ductal adenocarcinoma at Memorial Sloan Kettering Cancer Center and Garvan Medical Center undergoing surgery were recruited to participate in an Institutional Review Board-approved protocol. All patients who provided informed consent had samples collected, and study procedures were conducted in strict compliance with all ethical and institutional regulations. Although samples collected in short term survivor reflect genetic, histological, and clinical features of other short term PDAC survivors (ref # 5), they were collected through rapid autopsy which is a potential source of selection bias. Samples were collected at Memorial Sloan Kettering Cancer Center, Garvan Medical Center, and Johns Hopkins Hospital which may be a source of institutional bias.

Ethics oversight	Tissues from patients undergoing surgical resection at Memorial Sloan Kettering Cancer Center and Garvan Medical Center were collected under Institutional Review Board-approved study protocols.

Note that full information on the approval of the study protocol must also be provided in the manuscript.

Flow Cytometry

Plots

Confirm that:

☒ The axis labels state the marker and fluorochrome used (e.g. CD4-FITC).

☒ The axis scales are clearly visible. Include numbers along axes only for bottom left plot of group (a 'group' is an analysis of identical markers).

☒ All plots are contour plots with outliers or pseudocolor plots.

☒ A numerical value for number of cells or percentage (with statistics) is provided.

Methodology

Sample preparation	We purified peripheral blood mononuclear cells (PBMCs) from healthy donor buffy coats (New York Blood Center, New York, USA) and isolated T cells using a Pan-T cell isolation kit (Miltenyi Biotec, Germany). We activated T cells with CD3/CD28 beads (Thermo Fisher, MA, USA), IL7(3000 IU/mL), and IL15 (100 IU/mL) (Miltenyi Biotec), and transduced T cells with TCR constructs on day 2 post activation. We defined TCR transduced CD8+ T cells as live, CD3+, CD8+, mTCR+ cells. Full details are provided in the Methods.
Instrument	Flow cytometry was performed on an LSRFortessa (BD Biosciences; Catalog # 647177; Serial # H64717700135).
Software	Data were analyzed using FlowJo Software (version 10, Tree Star).
Cell population abundance	Representative cell abundance is indicated in Extended Data Figure 4b.
Gating strategy	The relevant gating strategy used is indicated in Extended Data Figure 4b.

☒ Tick this box to confirm that a figure exemplifying the gating strategy is provided in the Supplementary Information.
